# Inhibition of the deubiquitinase USP10 induces degradation of SYK

**DOI:** 10.1038/s41416-020-0731-z

**Published:** 2020-02-04

**Authors:** Jing Yang, Chengcheng Meng, Ellen Weisberg, Abigail Case, Ilaria Lamberto, Robert S. Magin, Sophia Adamia, Jinhua Wang, Nathanael Gray, Suiyang Liu, Richard Stone, Martin Sattler, Sara Buhrlage, James D. Griffin

**Affiliations:** 1 Department of Cancer Biology, Dana-Farber Cancer Institute, Harvard Medical School, Boston, 02215 MA USA; 2 Department of Biological Chemistry and Molecular Pharmacology, Harvard Medical School, Boston, MA USA; 3 Department of Medical Oncology, Dana-Farber Cancer Institute, Boston, MA USA; 4 Department of Medicine, Harvard Medical School, Boston, MA USA

**Keywords:** Acute myeloid leukaemia, Acute myeloid leukaemia

## Abstract

**Background:**

There is growing evidence that spleen tyrosine kinase (SYK) is critical for acute myeloid leukaemia (AML) transformation and maintenance of the leukemic clone in AML patients. It has also been found to be over-expressed in AML patients, with activating mutations in foetal liver tyrosine kinase 3 (FLT3), particularly those with internal tandem duplications (FLT3-ITD), where it transactivates FLT3-ITD and confers resistance to treatment with FLT3 tyrosine kinase inhibitors (TKIs).

**Methods:**

We have previously described a pharmacological approach to treating FLT3-ITD-positive AML that relies on proteasome-mediated FLT3 degradation via inhibition of USP10, the deubiquitinating enzyme (DUB) responsible for cleaving ubiquitin from FLT3.

**Results:**

Here, we show that USP10 is also a major DUB required for stabilisation of SYK. We further demonstrate that degradation of SYK can be induced by USP10-targeting inhibitors. USP10 inhibition leads to death of cells driven by active SYK or oncogenic FLT3 and potentiates the anti-leukemic effects of FLT3 inhibition in these cells.

**Conclusions:**

We suggest that USP10 inhibition is a novel approach to inhibiting SYK and impeding its role in the pathology of AML, including oncogenic FLT3-positive AML. Also, given the significant transforming role SYK in other tumours, targeting USP10 may have broader applications in cancer.

## Background

The non-receptor tyrosine kinase, SYK, is involved in several important signalling pathways.^1,2^ SYK is aberrantly activated in different hematologic malignancies, including B-cell lymphoma, chronic lymphocytic leukaemia (CLL) and mantle cell lymphoma, and acts as a driver oncogene in these disorders.^3–5^ SYK has also been shown to be activated and increased in FLT3-ITD-positive AML patients, is essential for transformation and has been implicated in resistance to targeted FLT3 TKIs.^6^ Highly activated SYK was observed to be enriched in AML patients, with more prevalence in FLT3-ITD-positive AML patients than those harbouring wild-type (wt) FLT3.^6^ SYK has also been shown to be associated with TKI resistance, and targeted FLT3 inhibition combined with SYK inhibition was shown to be more effective than FLT3 inhibition alone in FLT3-ITD-positive models of AML.^6,7^

Inhibition of FLT3-ITD-expressing AML cells with FLT3 kinase inhibitors, such as midostaurin, quizartinib, gilteritinib, sorafenib or crenolanib, has been shown to be effective in clinical trials to induce and extend remissions; however, many patients ultimately become resistant to TKI treatment. As an alternative, there is growing interest in developing strategies to reduce total levels of oncogenes such as FLT3-ITD through pharmacologically inducing protein degradation. The stability of many tyrosine kinases, including FLT3, is determined by ubiquitination and deubiquitination, each controlled by specific enzymes.^8–14^ We have recently shown that the major deubiquitinating enzyme for FLT3 is USP10. Specifically, either knockdown of USP10 or pharmacologic inhibition of this DUB resulted in sustained loss of FLT3-ITD protein and induction of leukemic cell death. These results suggested that it might be possible to treat TKI-resistant patients with USP10 inhibitors, leading to suppression of kinase activity and cell death.

DUBs are relative newcomers to the drug development scene. There is accumulating evidence that DUB enzymes contribute to cancer, at least in part through stabilisation of oncogenic proteins, and that potent and selective drug-like inhibitors can be developed. However, there are few examples of pharmacological DUB inhibitors inducing protein degradation for therapeutic benefit and even more fundamentally, the substrates of DUBs remain largely unknown. To ultimately achieve the vast therapeutic potential of being able to promote degradation of proteins with inherent or acquired resistance, studies focused on identifying and pharmacologically validating disease-relevant DUB substrates is needed. Here, we have considered the possibility that a similar strategy to target SYK in FLT3-mutant patients with AML might also be beneficial, since SYK is required for optimum transformation by FLT3-ITD.^6^ Interestingly, like FLT3, SYK is known to be a target of the E3 ligase c-CBL, and recent studies show that both FLT3 and SYK are regulated by ubiquitination and deubiquitination, which modulate both protein turnover and function.^15–18^ However, the DUBs that regulate SYK stability are unknown.

We found that targeted inhibition by small molecule inhibitors of USP10 impairs activated SYK-driven leukaemia cell proliferation and induces SYK protein degradation. USP10 forms a complex with FLT3-ITD and also physically associates with SYK, and through deubiquitination USP10 stabilises the levels of both proteins. Knockout (KO) and knockdown (KD) of USP10 destabilises SYK and shortens its protein half-life, with no effect on SYK mRNA levels. Our results present an alternative to SYK kinase inhibition and suggest a novel approach for treatment of some types of AML, including AML driven by oncogenic FLT3 or SYK.

## Methods

### Chemical compounds and biologic reagents

Midostaurin and crenolanib were purchased from Selleckchem (Houston, TX). DUB inhibitors HBX19818 and P22077 were purchased from Medchem Express. HBX19818 analogues, including C673-0105, C598-0556, C598-0563, C598-0466, C598-0515 and C598-0468, were purchased from ChemDiv. UPLC-MS analysis of all compounds was consistent with reported purity and molecular weight. All inhibitors were dissolved in DMSO to obtain a 10 mM stock solution. Serial dilutions were then made, to obtain final dilutions for cellular assays with a final concentration of DMSO between 0.2 and 0.5%.

### Cell lines and cell culture

Ba/F3 (interleukin [IL]-3-dependent murine pro-B) cells engineered to express SYK-TEL, FLT3-ITD and FLT3-ITD + SYK-TEL^6^ were provided by Dr Kimberly Stegmaier. Midostaurin-resistant Ba/F3 cell lines expressing FLT3 harbouring mutations in the ATP-binding pocket were developed as described previously.^19^ The human FLT3-ITD-postive AML line, MOLM14,^20^ was obtained from Dr Scott Armstrong, Dana Farber Cancer Institute (DFCI), Boston, MA. HEL cells were purchased from the American Type Culture Collection (ATCC) (Manassas, VA, USA).

As described previously,^7^ all cell lines used in this study were cultured with 5% CO_2_ at 37 °C, at a concentration of 2 × 10^5^ to 5 × 10^5^ in RPMI (Thermo Fisher Scientific, Waltham, MA) with 10% foetal bovine serum (FBS) and supplemented with 2% L-glutamine and 1% penicillin/streptomycin. Parental Ba/F3 cells were cultured in RPMI with 10% FBS and supplemented with 2% L-glutamine and 1% penicillin/streptomycin, as well as 20% WEHI (as a source of IL-3).

Human cell lines were submitted for cell line authentication and were authenticated within 6 months of manuscript preparation through cell line short tandem repeat (STR) profiling (DDC Medical, Fairfield, OH and Molecular Diagnostics Laboratory, Dana-Farber Cancer Institute). All cell lines tested matched >80% with lines listed in the ATCC or DSMZ Cell Line Bank STR. All cell lines were confirmed to be virus- and mycoplasma-free.

### Immunoblotting and immunoprecipitation

Protein lysate preparation, immunoblotting and immunoprecipitation were carried out as previously described.^21^

### Antibodies

The following antibodies were purchased from Cell Signaling Technology (Danvers, MA): AKT (rabbit, #9272), p44/42 MAPK (Erk1/2) (3A7) (mouse, #9107), Syk (D3Z1E) XP (rabbit mAb, #13198), beta-tubulin (rabbit, #2146), USP10 (D7A5) (rabbit mAb, #8501) and FES (rabbit, #2736) were used at 1:1000 for immunoblotting, and anti-GAPDH (14C10) (rabbit mAb, #2118) was used at 1:3000.

Flt-3/Flk-2 (C-20) (sc-479) and ubiquitin (P4D1) (sc-8017) were purchased from Santa Cruz Biotechnology, Inc. (Dallas, TX) and used at 1:1000 for immunoblotting. Anti-pTyr (mouse, clone 4G10) was purchased from MilliporeSigma (Burlington, MA) and was used at 1:1000 for immunoblotting in the presence of 4% BSA. Anti-HAUSP/USP7 antibody (ab4080) was purchased from Abcam (Cambridge, MA) and used at 1:1000 for immunoblotting.

### Proliferation studies and drug combination studies

The trypan blue exclusion assay has been previously described^21^ and was used to quantify cells prior to seeding for CellTiter-Glo assays. The CellTiter-Glo assay (Promega, Madison, WI) was used for proliferation studies and carried out according to manufacturer instructions. Cell viability is reported as percentage of control (DMSO-treated) cells, and error bars represent the standard deviation for each data point.

For drug combination studies, inhibitors were added simultaneously at fixed ratios to cells, and cell viability was expressed as the function of growth affected, drug-treated versus control cells. Data were analysed by Calcusyn software (Biosoft, Ferguson, MO and Cambridge, UK), which was utilised for synergy measurement and based on isobologram generation.^22^ This method utilises the median effect principle to quantify the effects of drug combinations to determine whether they yield effects together that are greater than that expected from a simple addition of their individual effects. After determining the ED_50_ or IC_50_ of each drug, combinations are studied in which the concentrations are multiples, or fractions, of the ED/IC_50_. Combination indices, values generated by the Calcusyn software, which are less than one indicates synergy, whereas values greater than one indicate antagonism. Calcusyn combination indices can be interpreted as follows: CI < 0.10 indicate very strong synergism; values 0.10–0.30 indicate strong synergism; values 0.30–0.70 indicate synergism; values 0.70–0.85 indicate moderate synergism; values 0.85–0.90 indicate slight synergism; values 0.90–1.10 indicate nearly additive effects; values 1.10–1.20 indicate slight antagonism; values 1.20–1.45 indicate moderate antagonism; values 1.45–3.30 indicate antagonism; values 3.3–10 indicate strong antagonism; values >10 indicate very strong antagonism.

### Crispr KO of USP10 and USP7

The Crispr KO assay was performed in CRISPR-CAS9 system. GFP-CAS9 expressing MOLM14 cells were generated by infecting MOLM14 with lentiviral GFP-CAS9 and GFP-sorted single cells were expanded to obtain clonal population. Cells were infected with Lentiviral gRNAs (target USP10 or USP7) (Thermo Fisher Scientific) and selected for puromycin resistance (1 µg/ml) after 72 h infection. Cells were collected after 3–5d of selection and protein as well as mRNA levels were determined by immunoblotting and Q-RT-PCR (*n* = 3).

### KD of USP10 by shRNA

pLKO.1puro lentiviral shRNA vector particles against *USP10* were purchased from Sigma–Aldrich (St. Louis, MO). Cells were incubated with the viral particles in the presence of 8 μg/ml Polybrene for 24 h, and the cells were selected with 1–2 μg/ml puromycin for 72 h. Following selection, cells were used for the studies described.

USP10 KD studies in MOLM14 cells: Viral particles were produced co-transfecting pLKO.1 containing shRNA or scramble (purchased from Sigma–Aldrich) together with psPAX2 (addgene#12260) and pMD2.G (addgene#12259), concentrated using LENTI-X concentrator (Clontech). MOLM14 cells were then infected in presence of 5 ug/ml polybrene and selection was started 48 h post infection using 1 ug/ml puromycin.

The sequences of the sgRNAs and shRNAs are shown as follows:

**Table:** 

USP7	T2	GAGTGATGGACACAACACCG
USP7	T3	GATTTCGCACAAAACACGGA
USP10	T2	TCCATCGACTGCCAGTACCC
USP10	T3	ATACTGTAGCTGGGGGTTCT
USP10	7431	CCGGCCTATGTGGAAACTAAGTATTCTCGAGAATACTTAGTTTCCACATAGGTTTTT
USP10	7432	CCGGCCCATGATAGACAGCTTTGTTCTCGAGAACAAAGCTGTCTATCATGGGTTTTT

### Quantitative real-time polymerase chain reaction (qPCR)

Following loss of USP10 via KD or KO, mRNA was extracted using the RNeasy Mini Kit (Qiagen) and converted to cDNA using SuperScript III reverse transcriptase (ThermoFisher). Real-time PCR was carried out in a 96-well plate using TaqMan probes and a 7500 FAST Real-Time PCR system (ThermoFisher). Relative gene expression was calculated by comparison to a GAPDH reference probe.

### Ubiquitin-AMC assay

Protein expression and purification. As described previously,^18^ a construct of human USP10 covering residues 376–798 in the pET28a vector was over-expressed in *E. coli* BL21 (DE3) in TB medium in the presence of 50 mg/ml of kanamycin. Cells were grown at 37 °C to an OD of 0.8, cooled to 17 °C, induced with 500 μM isopropyl-1-thio-D-galactopyranoside, incubated overnight at 17 °C, collected by centrifugation, and stored at −80 °C. Cell pellets were sonicated in buffer A (50 mM HEPES (pH 7.5), 300 mM NaCl, 10% glycerol, 10 mM Imidazole and 3 mM BME) and the resulting lysate was centrifuged at 30,000 × g for 30 min. Ni-NTA beads (Qiagen) were mixed with lysate supernatant for 30 min and washed with buffer A. Beads were transferred to an FPLC-compatible column and the bound protein was washed with 15% buffer B (50 mM HEPES (pH 7.5), 300 mM NaCl, 10% glycerol, 300 mM Imidazole and 3 mM BME) and eluted with 100% buffer B. Thrombin was added to the eluted protein and incubated at 4 °C overnight. The sample was then passed through a HiPrep 26/10 desalting column (GE Healthcare) pre-equilibrated with buffer A without imidazole, and the eluted protein was subjected to a second Ni-NTA step to remove His-tag and Thrombin. The eluent was concentrated and passed through a Superdex 200 10/300GL column (GE Healthcare) in a buffer containing 20 mM HEPES (pH 7.5), 150 mM NaCl, and 1 mM DTT. Fractions were pooled, concentrated to 20 mg/ml, and frozen at −80 °C. In vitro USP10 activity assay: Recombinant USP10, residues 376–798, was tested for its activity in a Ubiquitin-AMC assay in presence or absence of inhibitors. For this assay, 10 nM USP10 were pre-incubated with different concentrations of inhibitors or DMSO as a control in 50 mM HEPES pH 7.6, 0.5 mM EDTA, 11 uM ovalbumin, 5 mM DTT. The reaction was incubated for 6 h at room temperature prior to the addition of 2 uM Ubiquitin-AMC (Boston Biochem) substrate. The initial rate of the reaction was measured by collecting fluorescence data at 1-min interval over 30-min period using a Clariostar fluorescence plate reader at excitation and emission wavelength of 345 and 445 nm, respectively. The calculated initial rate values were plotted against inhibitor concentrations to determine IC50 values.

### HIS pull-downs

His-USP10 protein was purchased from R&D Systems (E-592-050), GST-SYK protein was purchase from Life Technologies Corporation (PV3857). GST protein was prepared following the standard protocol. For in vitro binding assays, purified GST or GST-SYK was incubated with His-USP10 coupled to NI-NTA beads for 3 h at 4 degree under cell-free conditions in NETT buffer (100 mM NaCl, 1 mM EDTA, 20 mM Tris-HCl, pH 8.0, 0.5% Triton-X-100) containing protease inhibitor (Thermo scientific, 78430) and 10 mM Imidazole. After washing five times, the bound proteins were boiled and separated by SDS-PAGE, then immunoblotted with indicated antibodies.

## Results

**Fig. 1:**
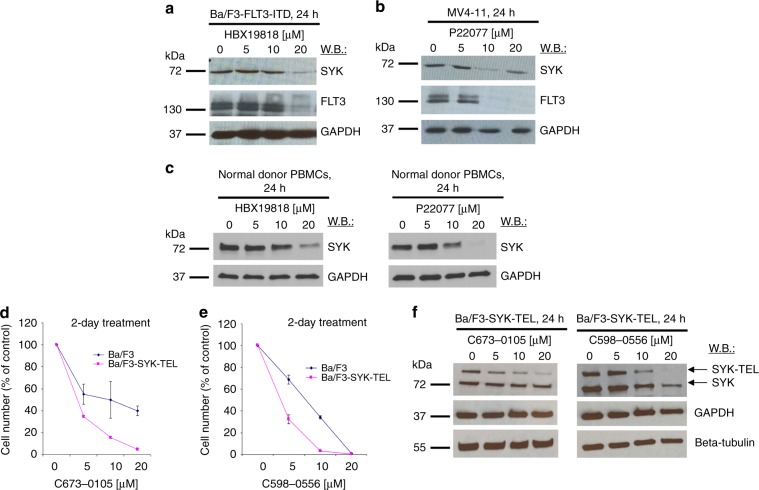
Small molecule inhibition of USP10 leads to degradation of SYK and targeted killing of activated SYK-driven cells. **a, b** Immunoblots: Effects of 24 h of treatment with HBX19818 or P22077 on FLT3 and SYK protein levels in Ba/F3-FLT3-ITD (**a**) or MV4-11 cells (**b**). **c** Immunoblots: Effects of 24 h of treatment with HBX19818 or P22077 on SYK protein levels in PBMCs from normal donors. **d, e** Proliferation assay: 2-day treatment of Ba/F3 versus Ba/F3-SYK-TEL cells with USP10-targeting inhibitors, C673-0105 (**d**) and C598-0556 (**e**). **f** Immunoblots: Effects of 24 h of treatment with C673-0105 and C598-0556 on SYK protein levels in Ba/F3-SYK-TEL cells.

Given our recent finding that USP10 deubiquitinates mutant FLT3,^18^ coupled with reported studies showing that FLT3 and SYK are interacting partners with each other and are targets of the same E3 ligase c-CBL,^6,7,15–17^ we hypothesised that USP10 may play a role in stabilisation of SYK. Early studies in our lab revealed wt SYK to be downregulated in FLT3-ITD-expressing Ba/F3 cells and MV4-11 cells in response to treatment with the DUB inhibitors, HBX19818 and P22077, both of which inhibit the activity of USP7 as well as USP10^18,23,24^ (Fig. 1a, b). We observed a similar drug-induced decrease in levels of SYK protein in cells co-expressing FLT3-ITD and tyrosine kinase domain (TKD) point mutations (Supplementary Fig. 1A, B). As expected, FLT3 protein levels were lower in all HBX19818- and P22077-treated, oncogenic FLT3-expressing cells, which we have previously shown is a consequence of inhibition of the DUB, USP10.^18^ In addition, HBX19818 and P22077 treatment led to a loss of SYK protein in peripheral blood mononuclear cells (PBMCs) from normal donors (Fig. 1c), indicating that USP10 regulates stability of SYK as well as constitutively activated SYK. Considering the potential significance of wt SYK as well as constitutively activated SYK in AML pathology and proposed role in drug resistance, we were interested in further exploring the loss of SYK protein in response to treatment with DUB inhibitors as a potential novel approach to treatment of AML.

We first wanted to confirm the role of USP10 in the observed decrease in SYK protein expression following treatment with HBX19818 and P22077, both of which inhibit the activity of USP7 as well as USP10. To accomplish this, we tested the ability of C598-0556 and C673-0105, two USP10-targeted structural analogues of HBX19818 displaying no USP7 inhibitory activity,^18^ to selectively kill cells driven by constitutively activated SYK (SYK-TEL). We observed a concentration-dependent decrease in proliferation of Ba/F3 cells over-expressing SYK-TEL and treated with C598-0556 and C673-0105; parental Ba/F3 cells were comparatively less sensitive (Fig. 1d, e). This inhibition of cell growth correlated with a targeted loss of constitutively activated SYK and wt SYK, in Ba/F3-SYK-TEL cells, which was observed after overnight to 24-h drug treatment (Fig. 1f and Supplementary Fig. 2A–D). The ability of C598-0556 to induce degradation of FLT3 in Ba/F3-FLT3-ITD cells was validated, along with P22077 and HBX19818 and select analogues (Supplementary Fig. 2E).

**Fig. 2:**
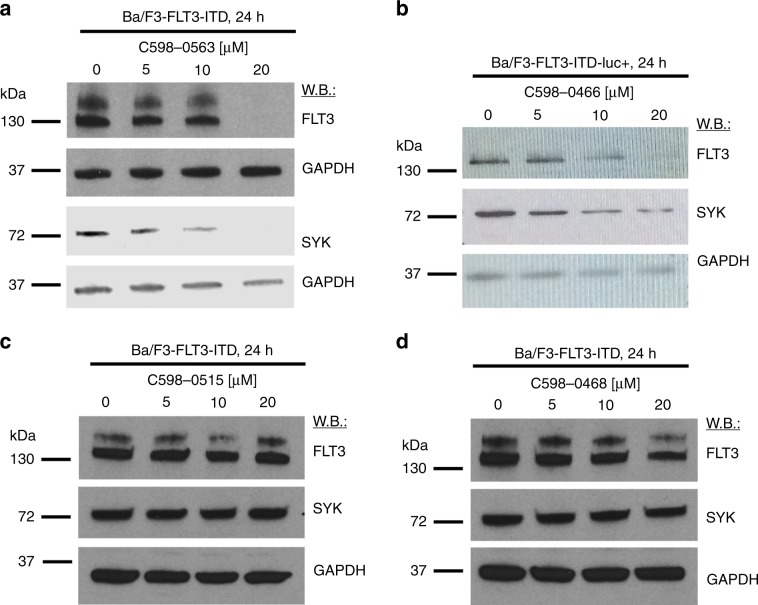
HBX19818 analogues have differential effects on SYK protein levels in Ba/F3-FLT3-ITD cells. Immunoblots: Effects of 24 h of treatment with C598-0563 (**a**), C598-0466 (**b**), C598-0515 (**c**) and C598-0468 (**d**).

To further assess the potential role of USP10 in stabilisation of SYK, we correlated the USP10 inhibitory activity of a panel of HBX19818 analogues, as previously determined in a biochemical assay^18^ (Supplementary Fig. 3 A), with influence on SYK protein levels. Good correlation among these parameters was observed, with HBX19818 analogues, such as C598-0563 and C598-0466, showing activity against USP10 in the biochemical assay and strongly inducing degradation of SYK protein in Ba/F3-FLT3-ITD cells (Fig. 2a, b and Supplementary Fig. 3A, B). In addition to degrading wt SYK in Ba/F3-FLT3-ITD cells, C598-0563 also leads degradation of activated SYK in Ba/F3-SYK-TEL cells (Supplementary Fig. 3C). Conversely, HBX19818 analogues, such as C598-0515 and C598-0468, showed little activity in the biochemical assay against USP10 and also induced no degradation of SYK in Ba/F3-FLT3-ITD cells (Fig. 2c, d and Supplementary Fig. 3A). These results support USP10 as the relevant target of HBX19818 and its analogues.

**Fig. 3:**
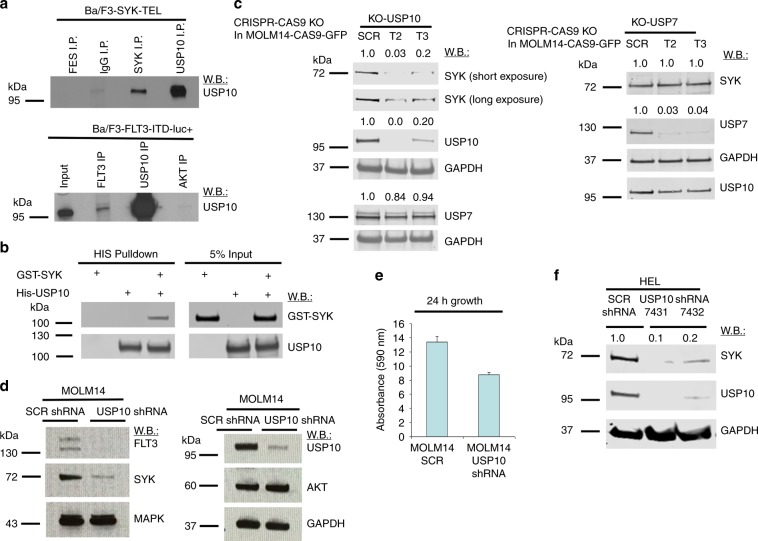
USP10 and SYK physically associate and genetic knockdown or knockout of USP10 leads to SYK degradation. **a** Immunoprecipitation/immunoblots: Co-immunoprecipitation of SYK protein and USP10 protein in Ba/F3-SYK-TEL cells and co-immunoprecipitation of FLT3 protein and USP10 protein in Ba/F3-FLT3-ITD-luc + cells. **b** Purified GST-SYK protein was incubated with His-USP10 coupled to NI-NTA beads. Proteins retained on NI-NTA beads were then blotted with indicated antibodies. **c** Immunoblots: Effects of Crispr knockout of USP10 on SYK protein levels in MOLM14-Cas9-GFP #15 monoclonal cells and effects of Crispr knockout of USP7 on SYK protein levels in MOLM14-Cas9-GFP #8 monoclonal cells. Shown in **c** are densitometry values for SYK, USP10 and USP7 shown relative to GAPDH. **d** Immunoblots: Effects of USP10 KD on SYK, FLT3, MAPK and AKT expression in MOLM14 cells. **e** Measurement of MOLM14 SCR shRNA and MOLM14 USP10 shRNA cell growth by CellTiter Glo following 24 h after seeding. Error bars represent standard deviations. **f** USP10 shRNA KD in HEL cells. Shown in **f** are densitometry values for SCR versus USP10 shRNAs, shown relative to GAPDH.

To investigate whether SYK and USP10 physically associate in a protein complex, we performed co-immunoprecipitation experiments. We demonstrate a potential physical association between SYK and USP10, as both proteins co-immunoprecipitate in Ba/F3-SYK-TEL cells (a Fes immunoprecipitation was included as a control for nonspecific binding) (Fig. 3a, upper panel and Supplementary Fig. 4A, B). Our current data also support our previous observation that FLT3 and USP10 bind to one another,^18^ as FLT3 and USP10 co-immunoprecipitate in Ba/F3-FLT3-ITD cells (an AKT immunoprecipitation was included as a control for nonspecific binding) (Fig. 3a, lower panel). To determine whether the USP10 could interact with SYK directly, we performed an in vitro binding assay with purified recombinant His-USP10 and GST-SYK proteins. Purified His-USP10 was able to interact with GST-SYK under cell-free conditions, suggesting a direct interaction between USP10 and SYK (Fig. 3b and Supplementary Fig. 4C). Finally, as has been previously reported,^18^ our data suggest that SYK and FLT3 bind to one another, as both proteins co-immunoprecipitate in Ba/F3 cells co-expressing FLT3-ITD and SYK-TEL (Supplementary Fig. 4D).

To validate the role of USP10 in the stabilisation of SYK protein, we performed genetic studies using both CRISPR and shRNA. USP10 CRISPR knockout (KO) was carried out with MOLM14-Cas9-GFP monoclonal cells. We observed less SYK protein in USP10 KO cells as compared to SCR KO controls; in contrast, USP7 KO did not affect levels of SYK (Fig. 3c and Supplementary Fig. 5A). Similar effects of USP10 KO and USP7 KO on SYK protein levels were observed in wt FLT3-expressing HEL-Cas9-GFP cells, which were investigated to determine if SYK is a substrate of USP10 in both wt and mutant FLT3-expressing AML (Supplementary Fig. 5B). We also performed USP10 KD in MOLM14 cells; loss of FLT3 and SYK protein was observed in USP10 KD cells; however, there was no apparent decrease observed in protein levels of MAPK and AKT (Fig. 3d and Supplementary Fig. 6). The loss of FLT3 and SYK correlated with a slower rate of growth of cells following 24 h after seeding of cells (Fig. 3e). USP10 KD was additionally performed in HEL cells and led to a decrease in levels of SYK protein (Fig. 3f).

**Fig. 4:**
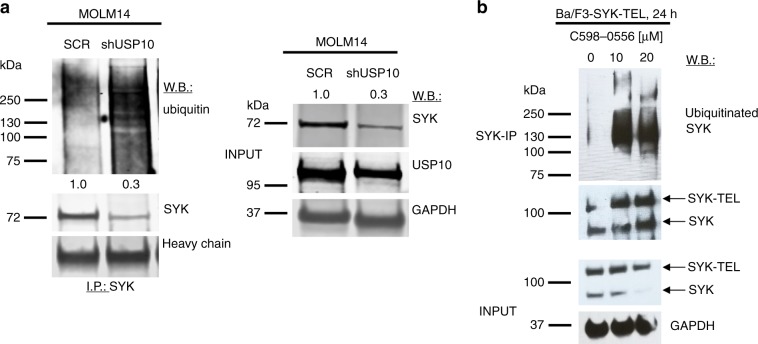
USP10 KD or small molecule inhibition leads to ubiquitination and degradation of SYK and increased total cellular ubiquitination. **a** Immunoprecipitation (I.P.)/western blotting (W.B.): Effect of USP10 KD on ubiquitination and degradation of SYK. Shown are densitometry values for SCR versus shUSP10, shown relative to GAPDH. **b** I.P./W.B.: Effect of C598-0556 on inducing ubiquitination and degradation of SYK. For experiments shown in Fig. 4a and Fig. 4b, a SYK I.P. was performed on SCR versus USP10 KD MOLM14 cells (Fig. 4a) and on untreated versus C598-0556-treated Ba/F3-SYK-TEL cells (Fig. 4b), followed by a ubiquitin W.B.

SYK has been demonstrated to undergo ubiquitination.^25^ To establish whether the loss of SYK in USP10 inhibitor-treated cells was mediated by ubiquitination, we compared protein lysates from MOLM14 cells characterised by genetic KD of USP10. A SYK I.P. and ubiquitin immunoblot showed a higher level of ubiquitinated SYK in the USP10 KD cells as compared to SCR controls, as well as degraded SYK protein (Fig. 4a). Consistent with this, treatment of Ba/F3-SYK-TEL cells with C598-0556 also led to SYK degradation and an increase in SYK ubiquitination levels, as compared to DMSO-treated control cells (Fig. 4b). These results support the notion that the USP10 inhibition leads to ubiquitin-mediated SYK degradation.

**Fig. 5:**
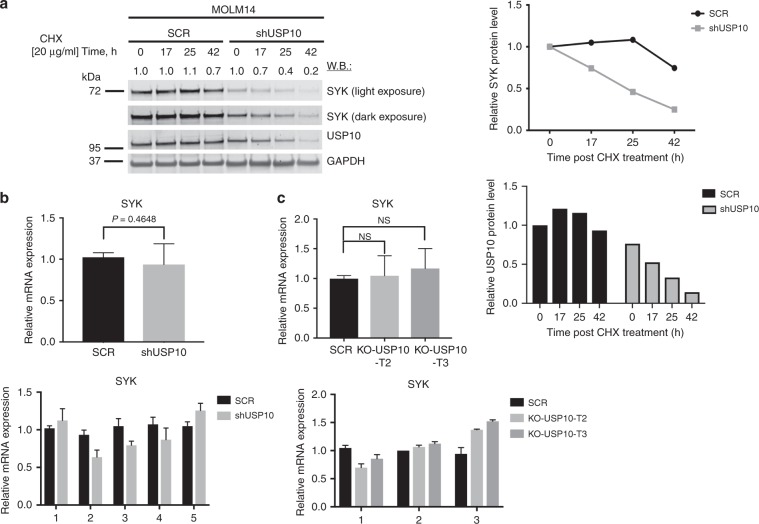
Loss of USP10 shortens the half-life of SYK protein with no effect on SYK mRNA transcription. **a** Effects of USP10 KD on the half-life of SYK in MOLM14 cells as compared to SCR control. This experiment is representative of two independent experiments for which similar results were observed (*n* = 2). Shown are densitometry values for SCR versus shUSP10, shown relative to GAPDH. **b** Effect of USP10 KD on SYK transcription in MOLM14 cells. Shown is SYK expression relative to GAPDH expression. This experiment is representative of five independent experiments for which similar results were observed (*n* = 5). **c** Effect of USP10 KO on SYK transcription in MOLM14 cells. Shown is SYK expression relative to GAPDH expression. This experiment is representative of three independent experiments for which similar results were observed (*n* = 3).

To further establish whether loss of USP10 leads to loss of SYK at the level of protein and is not due to suppression of transcription, we first analysed the half-life of SYK in USP10 KD versus SCR KD control MOLM14 cells and found USP10 KD to considerably shorten the half-life of SYK (Fig. 5a and Supplementary Fig. 7). Secondly, qPCR analysis confirmed that the reduction in SYK levels in both USP10 KD MOLM14 cells and USP10 KO MOLM14 cells occurred at the protein level only (Fig. 5b, c).

Highly activated SYK has been observed to occur more frequently in AML patients expressing oncogenic FLT3 as opposed to its wt counterpart, transactivates FLT3-ITD and, when over-expressed, contributes to transformation and resistance to FLT3 inhibitors.^6^ Having already shown that USP10 inhibitors, such as HBX19818 and P22077, degrade SYK in FLT3-ITD-expressing Ba/F3 cells and MV4-11 cells (Fig. 1a, b), we further demonstrated the ability of USP10-targeting inhibitors, C598-0556 and C673-0105, to kill Ba/F3 cells specially engineered to co-express FLT3-ITD and constitutively activated SYK-TEL in a concentration-dependent manner (Supplementary Fig. 8A); cell killing correlated with degradation of SYK and FLT3-ITD in these cells (Supplementary Fig. 8B).

**Fig. 6:**
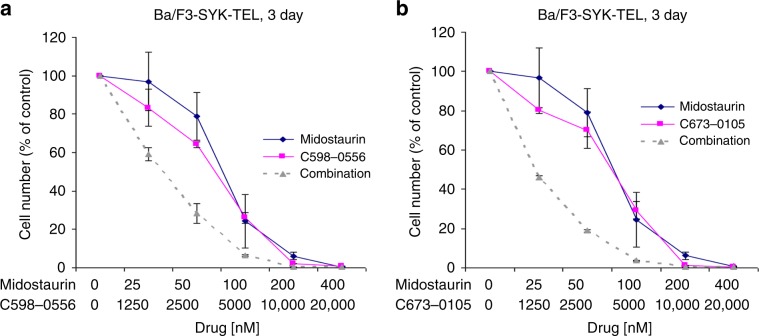
Midostaurin potentiates the effects of USP10 inhibitors against Ba/F3-SYK-TEL cells. (**a**–**b**) Proliferation study: Effects of the combination of midostaurin and C598-0556 (left panel) and midostaurin and C673-0105 (right panel) on growth of Ba/F3-SYK-TEL cells following 3 days of treatment.

The combined inhibition of FLT3 by a targeted FLT3 inhibitor, such as quizartinib, and inhibition of SYK has been shown to be more efficacious than inhibition with a FLT3 inhibitor alone.^6^ As these results support the idea that potentiation of FLT3 inhibition can be achieved by suppression of SYK, we hypothesised that removal of SYK protein by DUB inhibitor-induced degradation might similarly augment the effects of FLT3 inhibition in leukemic cells. FLT3 inhibitors and USP10-targeting inhibitors synergised when combined and used to treat Ba/F3 cells driven by FLT3-ITD only or activated SYK only, or a combination of FLT3-ITD and activated SYK (Fig. 6, Supplementary Fig. 9 and Table 1). In contrast, there was no positive combination effect observed when midostaurin plus the USP10-targeting inhibitors or crenolanib plus the USP10-targeting inhibitors were tested against parental Ba/F3 cells (Supplementary Fig. 9 and Table 1). These results support our earlier findings, suggesting synergistic effects between the parent compound, HBX19818, combined with crenolanib or midostaurin against Ba/F3-FLT3-ITD cells or FLT3-ITD-positive MOLM14 cells.^18^ Our findings also suggest that the combination of a FLT3 inhibitor and a USP10-targeting inhibitor may have broader therapeutic implications, offering potential clinical benefit for patients harbouring leukemic cells driven by oncogenic FLT3 or oncogenic SYK, or both. Transactivation of FLT3-ITD by SYK has been reported, and over-expression or hyperactivation of SYK contributes to drug resistance in AML. The observed synergy between FLT3 inhibition and SYK suppression via USP10 inhibition and consequent SYK protein degradation could offer a novel therapeutic approach to override drug resistance in this context.

**Table 1:** Combination indices generated by Calcusyn software for crenolanib ± C598-0556, crenolanib ± C673-0105, midostaurin ± C598-0556 and midostaurin ± C673-0105 tested against Ba/F3-SYK-TEL, Ba/F3-FLT3-ITD and Ba/F3-FLT3-ITD + SYK-TEL cells.

Cell line	Days of treatment	Drug combination	ED25	ED50	ED75	ED90	CI value
Ba/F3-SYK-TEL	2 days	crenolanib + C598-0556	0.92	1.00	1.08	1.19351	
Ba/F3-SYK-TEL	2 days	crenolanib + C673-0105	0.83	0.90	0.99	1.09124	
Ba/F3-SYK-TEL	3 days	crenolanib + C598-0556	0.93	1.03	1.15	1.29099	0.00
Ba/F3-SYK-TEL	3 days	crenolanib + C673-0105	0.68	0.77	0.90	1.0444	0.25
Ba/F3-SYK-TEL	3 days	midostaurin + C598-0556	0.94	1.01	1.08	1.15254	0.50
Ba/F3-SYK-TEL	2 days	midostaurin + C673-0105	0.80	0.88	0.95	1.03945	0.75
Ba/F3-SYK-TEL	3 days	midostaurin + C598-0556	0.89	0.95	1.01	1.07668	1.00
Ba/F3-SYK-TEL	3 days	midostaurin + C673-0105	0.67	0.74	0.81	0.90019	1.25
Ba/F3-FLT3-ITD	2 days	crenolanib + C598-0556	0.48	0.55	0.63	0.72655	1.50
Ba/F3-FLT3-ITD	2 days	crenolanib + C673-0105	0.55	0.62	0.71	0.81216	
Ba/F3-FLT3-ITD	3 days	crenolanib + C598-0556	0.31418	0.37855	0.4561	0.54956	
Ba/F3-FLT3-ITD	3 days	crenolanib + C673-0105	0.36445	0.45081	0.55764	0.68978	
Ba/F3-FLT3-ITD	2 days	midostaurin + C598-0556	0.54678	0.65506	0.78506	0.9412	
Ba/F3-FLT3-ITD	2 days	midostaurin + C673-0105	0.57086	0.68038	0.81145	0.96839	
Ba/F3-FLT3-ITD	3 days	midostaurin + C598-0556	0.63815	0.73788	0.8532	0.98654	
Ba/F3-FLT3-ITD	3 days	midostaurin + C673-0105	0.78463	0.85346	0.93045	1.01679	
Ba/F3-FLT3-ITD + SYK-TEL	2 days	crenolanib + C598-0556	0.67777	0.76846	0.87128	0.98786	
Ba/F3-FLT3-ITD + SYK-TEL	2 days	crenolanib + C673-0105	1.01059	1.04929	1.09134	1.13705	
Ba/F3-FLT3-ITD + SYK-TEL	3 days	crenolanib + C598-0556	0.80774	0.90974	1.02484	1.15475	
Ba/F3-FLT3-ITD + SYK-TEL	3 days	crenolanib + C673-0105	0.79295	0.88725	0.99281	1.11095^a^	
Ba/F3-FLT3-ITD + SYK-TEL	2 days	midostaurin + C598-0556	0.78454	0.94376	1.1353	1.3657^a^	
Ba/F3-FLT3-ITD + SYK-TEL	2 days	midostaurin + C673-0105	0.65125	0.77988	0.93409	1.11899	
Ba/F3-FLT3-ITD + SYK-TEL	3 days	midostaurin + C598-0556	0.58832	0.70615	0.84757	1.01732	
Ba/F3-FLT3-ITD + SYK-TEL	3 days	midostaurin + C673-0105	0.81731	0.9078	1.01196	1.13232	

Proliferation assays/combination studies were carried out for 2 and 3 days, respectively.

^a^Combination indices were calculated based on four data points. Combination indices for all other studies were calculated based on five data points.

## Discussion

Hyperactivated SYK is a driver oncogene in a number of hematologic malignancies. Among these are B cell-derived lymphomas, where SYK plays a major role in promoting cell survival through mediation of tonic and chronic signalling through the BCR.^3–5,26,27^ In addition, the majority of B cell acute lymphocytic leukaemia (B-ALL) cases have been found to be characterised by expression of constitutively activated SYK and are responsive to SYK inhibitor treatment.^28,29^ SYK has also been implicated as a major player in the pathogenesis of AML, and specifically in transformation and TKI inhibitor resistance of AML cells harbouring FLT3-ITD.^6,30^ Although SYK is rarely mutated in cancer,^31^ chromosomal translocations involving the SYK gene have been identified, such as TEL-SYK in myelodysplastic syndrome^32^ and ITK-SYK, found in T-cell lymphoproliferative diseases.^33^

The involvement of SYK in transformation of oncogenic FLT3-expressing AML supports the idea that degradation of SYK may potentially provide clinical benefit for this subset of AML patients. The small percentage of AML patients that harbour mutated SYK could potentially benefit from this novel therapeutic strategy as well. Therapeutic targeting of SYK by promoting its degradation as opposed to inhibition of its kinase activity is potentially beneficial for overcoming resistance to TKIs, which can arise from such mechanisms as target protein amplification or development of point mutations. In addition, induction of SYK degradation may prove more efficacious than SYK kinase inhibition by simultaneously inhibiting both enzymatic and scaffolding functions of SYK.

We have previously shown that USP10 stabilises oncogenic FLT3 and targeting of USP10 leads to death of mutant FLT3-positive cells.^18^ Here, we validated USP10 as a DUB that stabilises SYK by showing that targeted loss of USP10 by genetic KO or KD leads to ubiquitin-mediated degradation of SYK protein in AML cells, with no effect on SYK transcription. In addition, we show that USP10 inhibition potentiates the effects of FLT3 inhibition in cells driven by activated SYK. It is possible that other DUBs exist that also stabilise SYK and have not yet been identified; however, it is evident from our studies that USP10 is sufficient for SYK stabilisation.

Both FLT3 and SYK are substrates of the ubiquitin E3 ligase, c-CBL.^34^ Higher FLT3 protein levels due to lack of regulation by functional CBL has been associated with aberrant FLT3 signalling,^35^ and previous work^6,7^ suggest that this could potentially lead to transactivation of SYK, together contributing to cellular transformation. Direct targeting of CBL is challenging and unlikely to be beneficial due to mutations of CBL being inactivating. Further, patients with myeloid neoplasms having CBL mutations generally have a poor prognosis and so novel therapeutic strategies are urgently needed.

It is well established that SYK plays a key role in the survival of a number of different types of hematologic malignancies, with much attention focused on SYK promotion of growth of malignancies of B cell origin and survival attenuated by inhibition or removal of SYK. Importantly, however, SYK has been implicated in the transformation of a variety of non-hematologic malignancies as well, and therefore novel approaches to target SYK through such tactics as protein destabilisation could potentially have wide-reaching clinical relevance and application. Activation of SYK is a consequence of its recruitment to an immune recognition receptor, through binding of its SH2 domains to a phosphorylated immunoreceptor tyrosine-based activation motif (ITAM).^36^ Several types of cancer, including nasopharyngeal carcinoma and breast cancer, are characterised by abnormal expression of ITAM-containing proteins and hyperactivation of SYK.^37,38^ The pro-survival activity of SYK has also been implicated in such malignancies as KRAS-dependent solid tumours and retinoblastoma.^39,40^ SYK is also a potential oncogenic driver in subset of small cell lung cancer, and high expression in squamous cell carcinomas of the head and neck is linked to metastasis and a poor prognosis.^41,42^ The prospective importance of SYK in tumorigenesis is further suggested by its generally higher transcript levels in transformed- as opposed to normal-tissues, including that of the ovary, colon, kidney and brain.^43^

Our findings support the idea of using small molecule induce degradation of SYK as an alternative to SYK kinase inhibition, alone or combined with FLT3 inhibition for the treatment of AML characterised by FLT3-ITD and transformed by SYK. The use of USP10 inhibition to degrade SYK may additionally be explored as a potential treatment approach for other malignancies for which SYK plays an important role.

## Supplementary information

Supplementary information

## Data Availability

The authors declare that the main data supporting the findings of this study are available within the article and its supplementary information files.
