# Supplementary material for: Inhibition of the deubiquitinase USP10 induces degradation of SYK
Source: Br J Cancer. 2020 Feb 4;122(8):1175–84. doi: 10.1038/s41416-020-0731-z (PMC7156412; doi:10.1038/s41416-020-0731-z)
Supplement: Supplementary file 1 — Supplementary information [file 41416_2020_731_MOESM1_ESM.pdf]

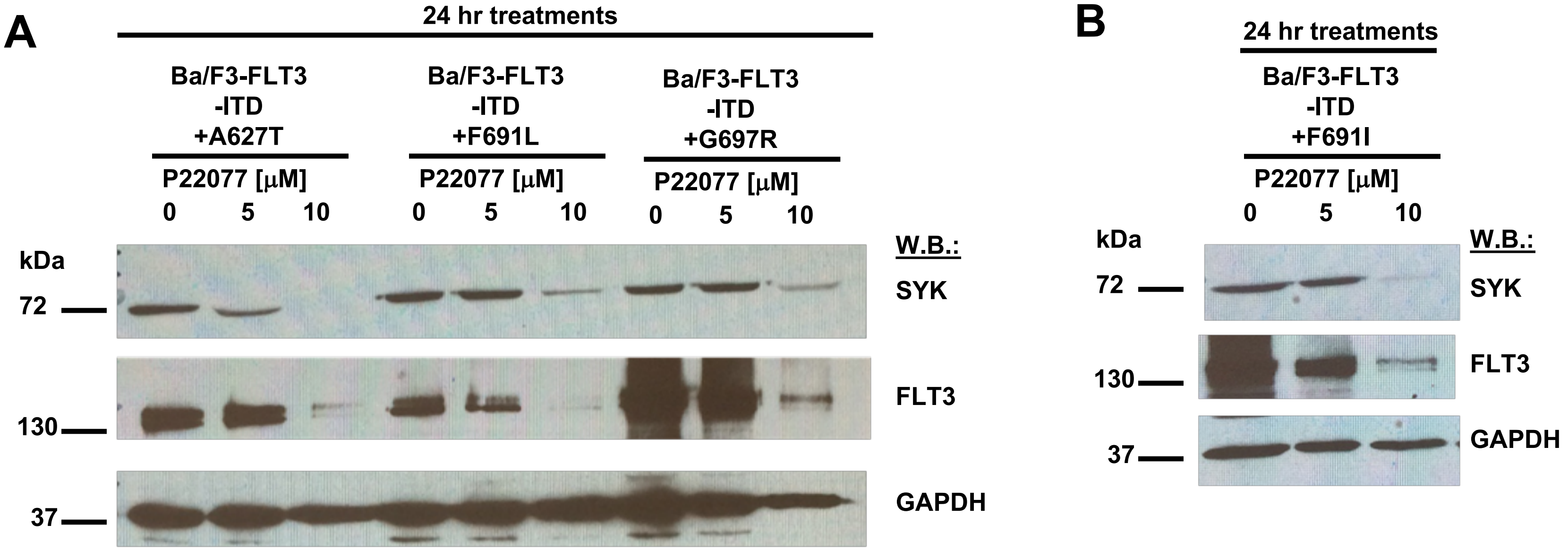

Supplementary Figure 1

Supplementary Figure 2

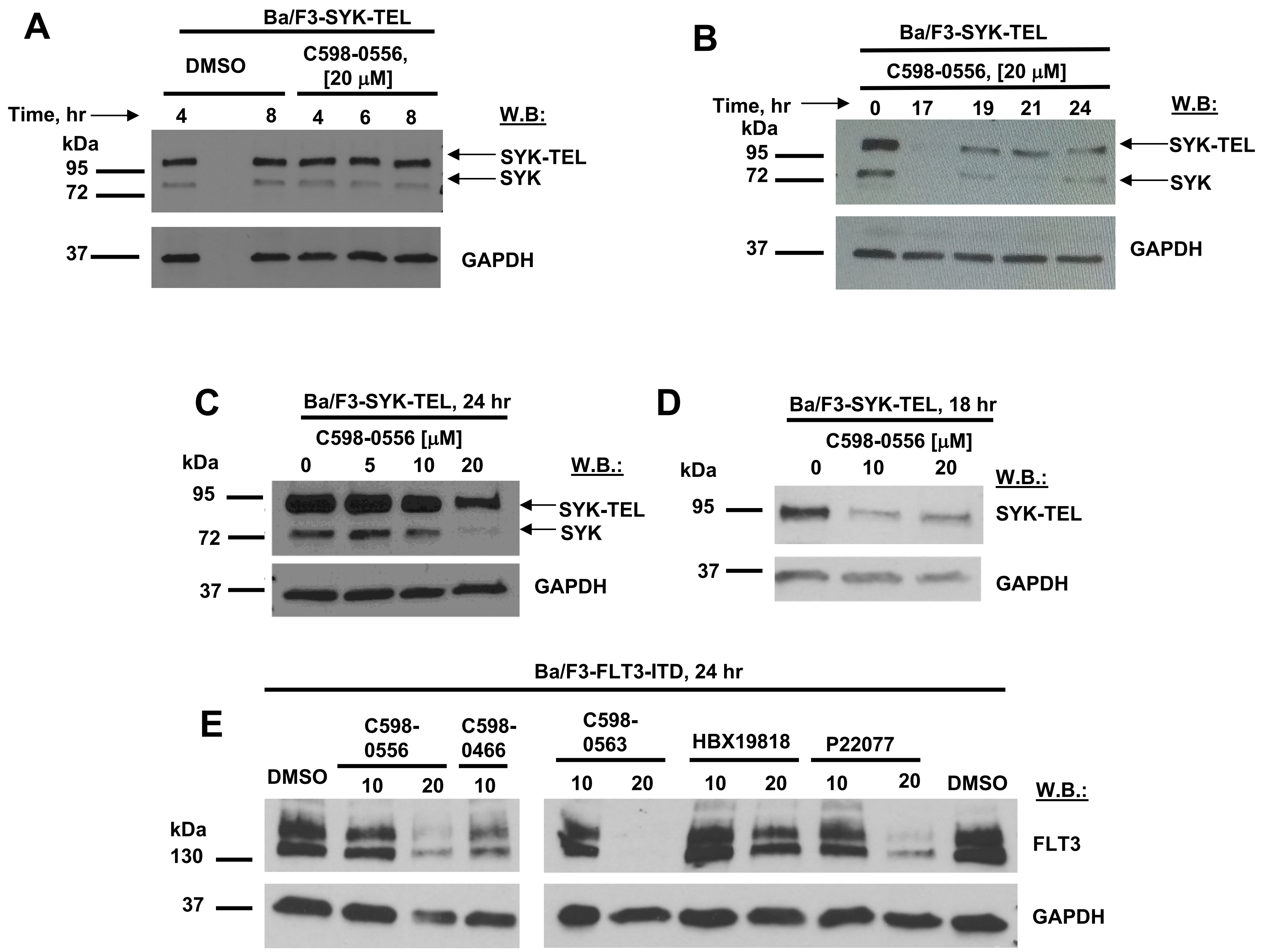

A

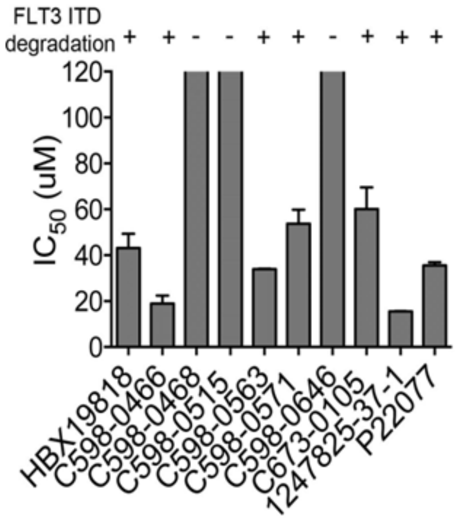

Compound	IC_{50} (uM) ± SEM (n)
HBX19818	43 ± 6.2 (3)
C598-0466	19 ± 3.5 (3)
C598-0468	>>100 (3)
C598-0515	>>100 (4)
C598-0563	34 ± 0.24 (2)
C598-0571	54 ± 6.0 (2)
C598-0646	>>100 (3)
C673-0105	60 ± 9.5 (2)
P22077	36 ± 1.4 (2)
1247825-37-1	15 ± 0.11 (2)

B

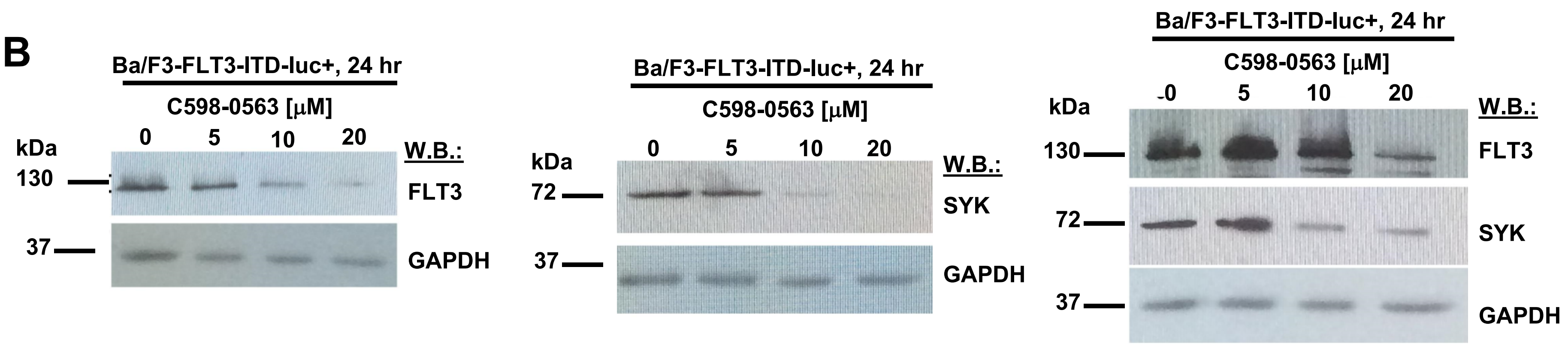

C

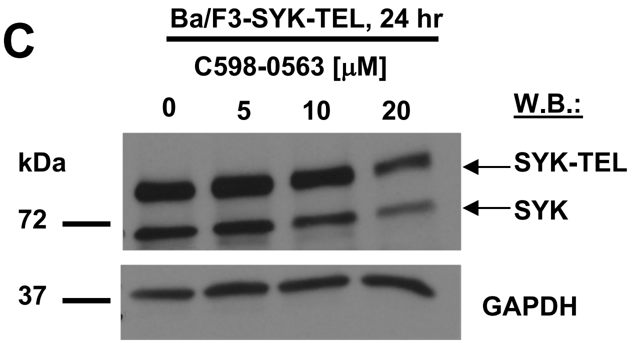

Supplementary Figure 3

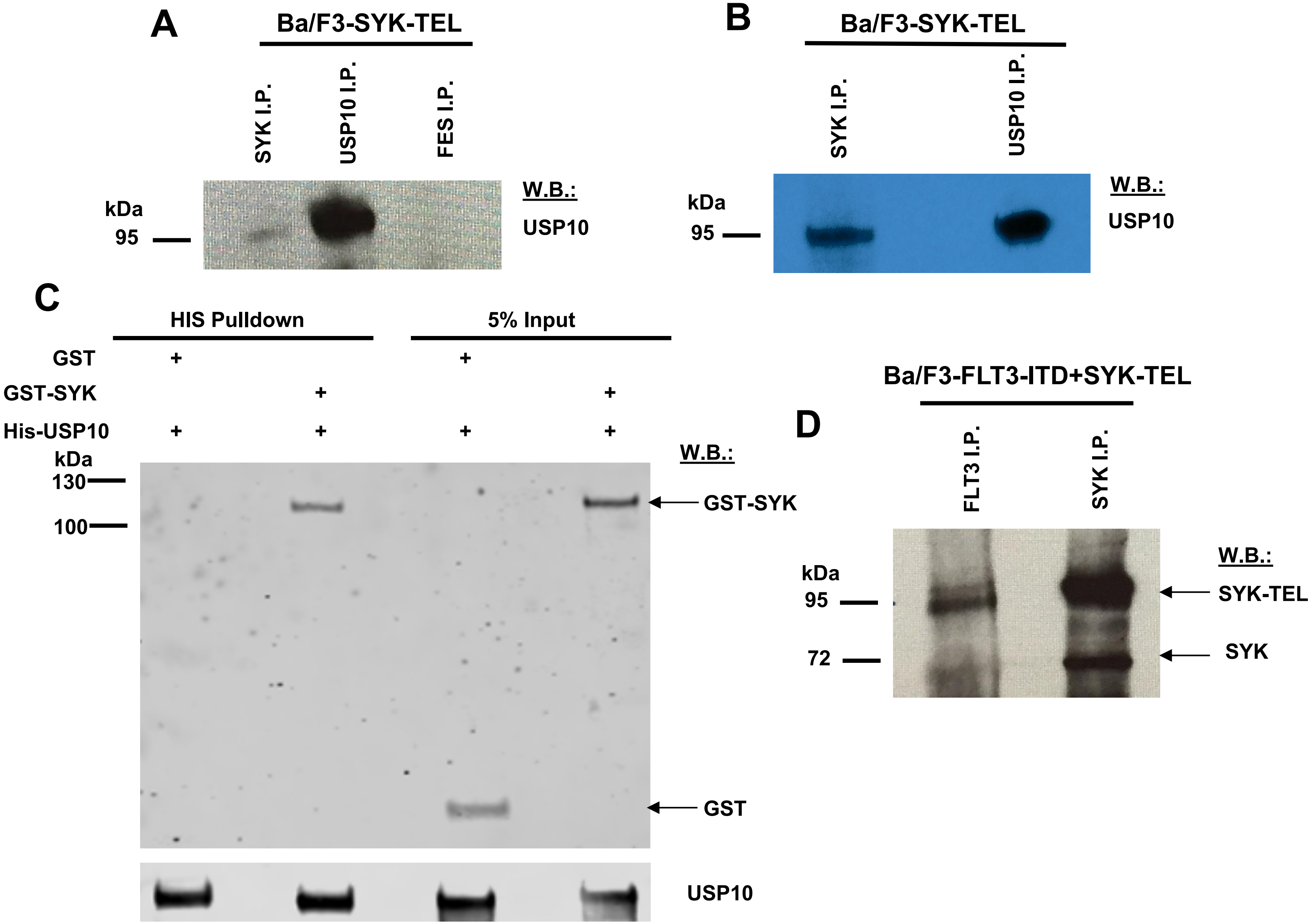

Supplementary Figure 4

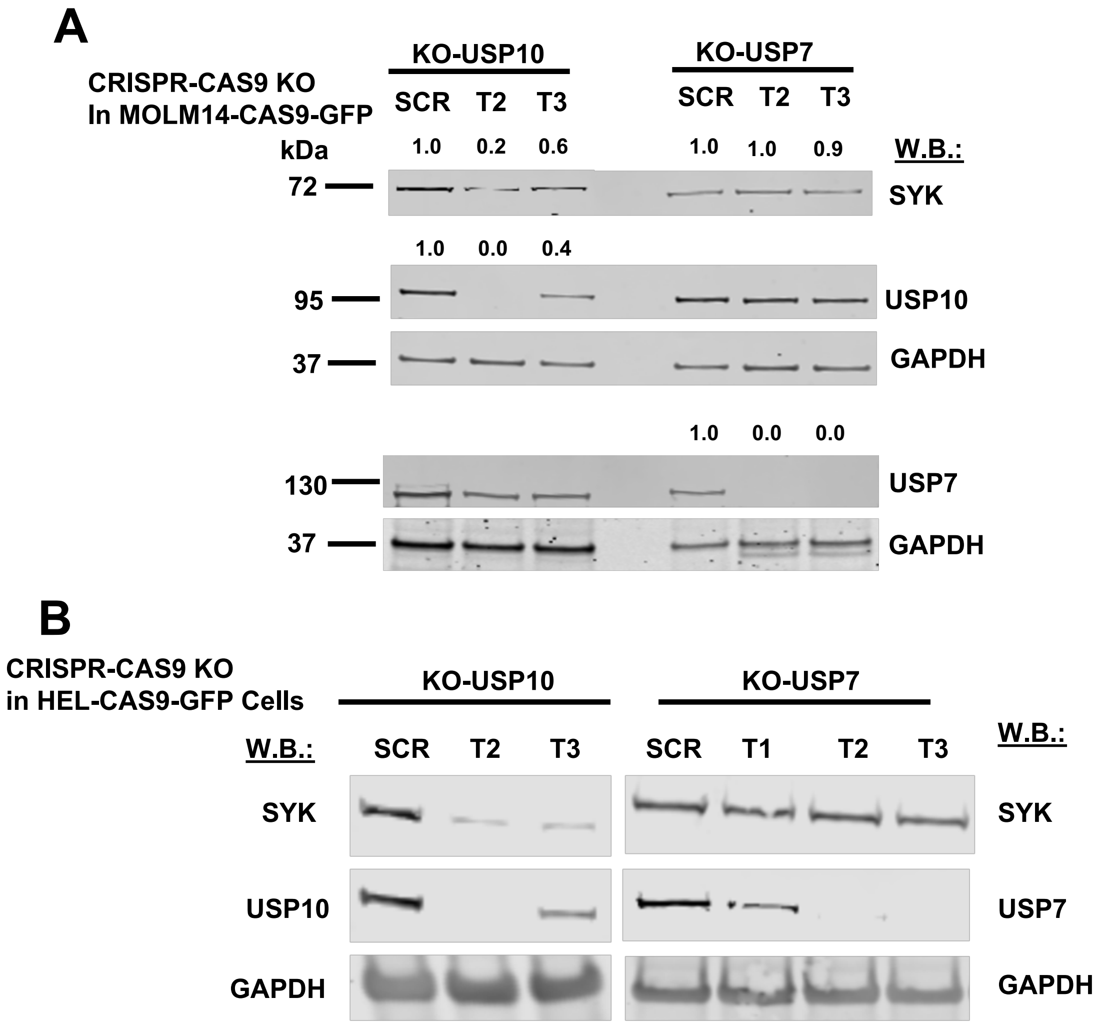

Supplementary Figure 5

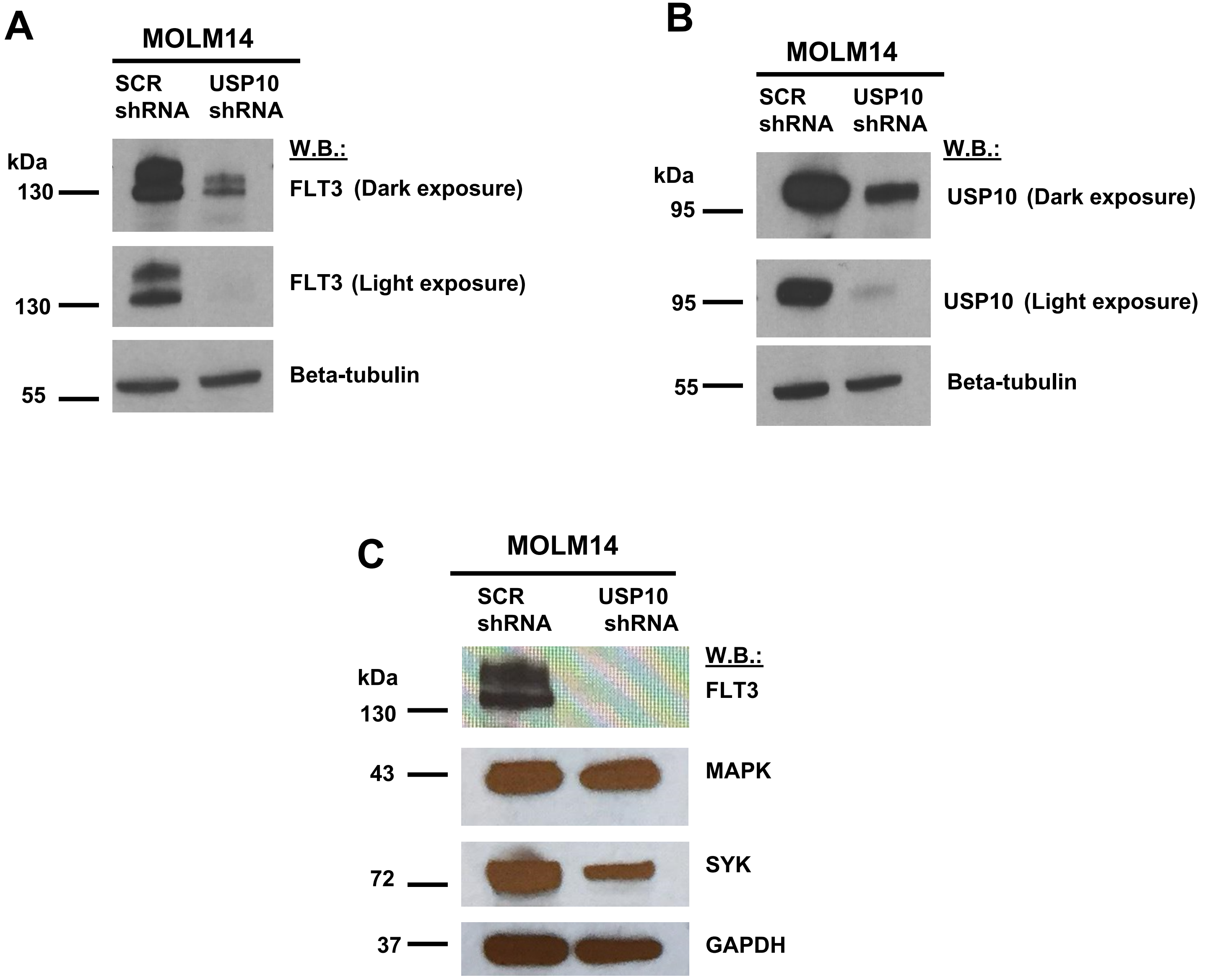

Supplementary Figure 6

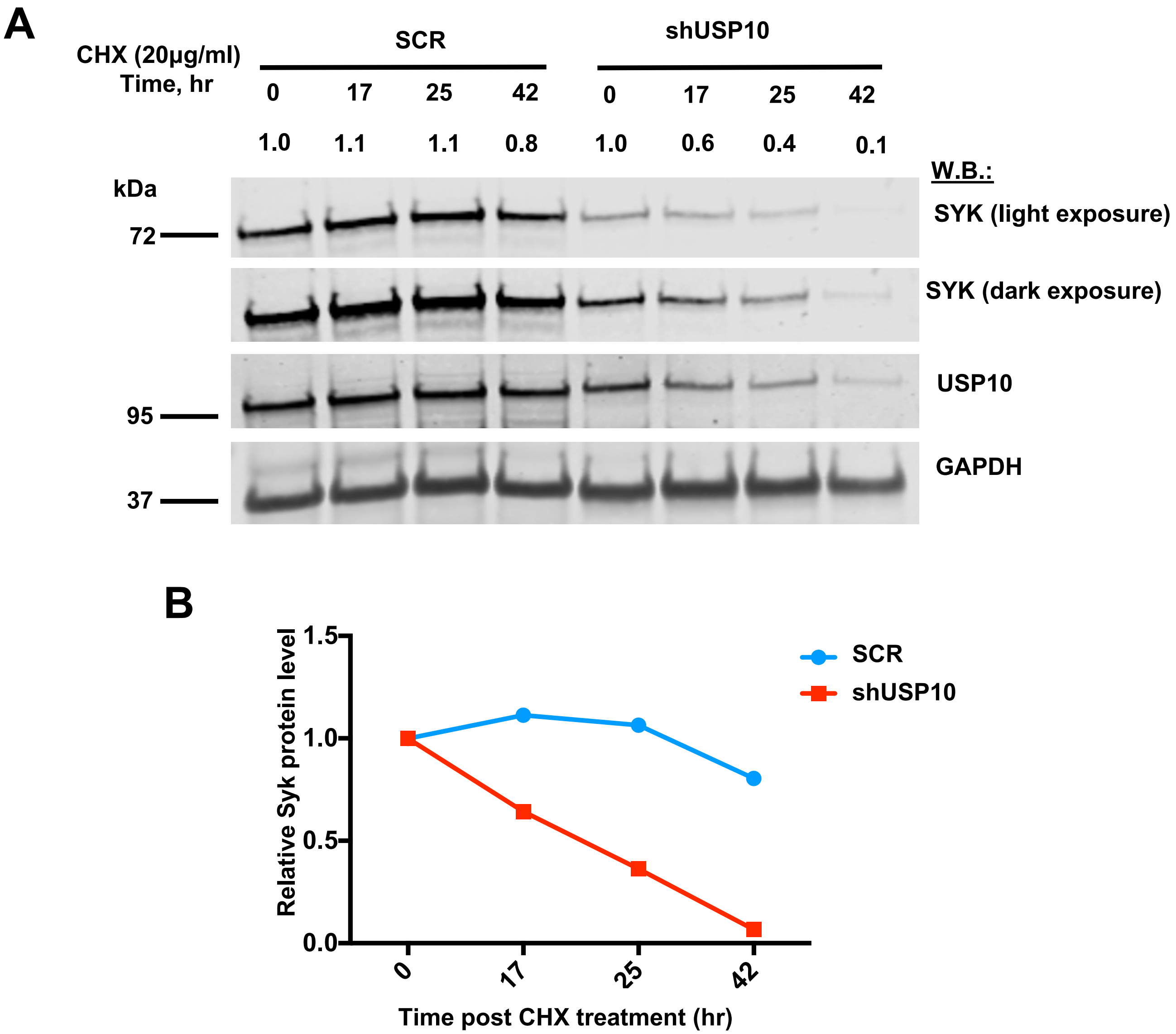

Supplementary Figure 7

Supplementary Figure 8

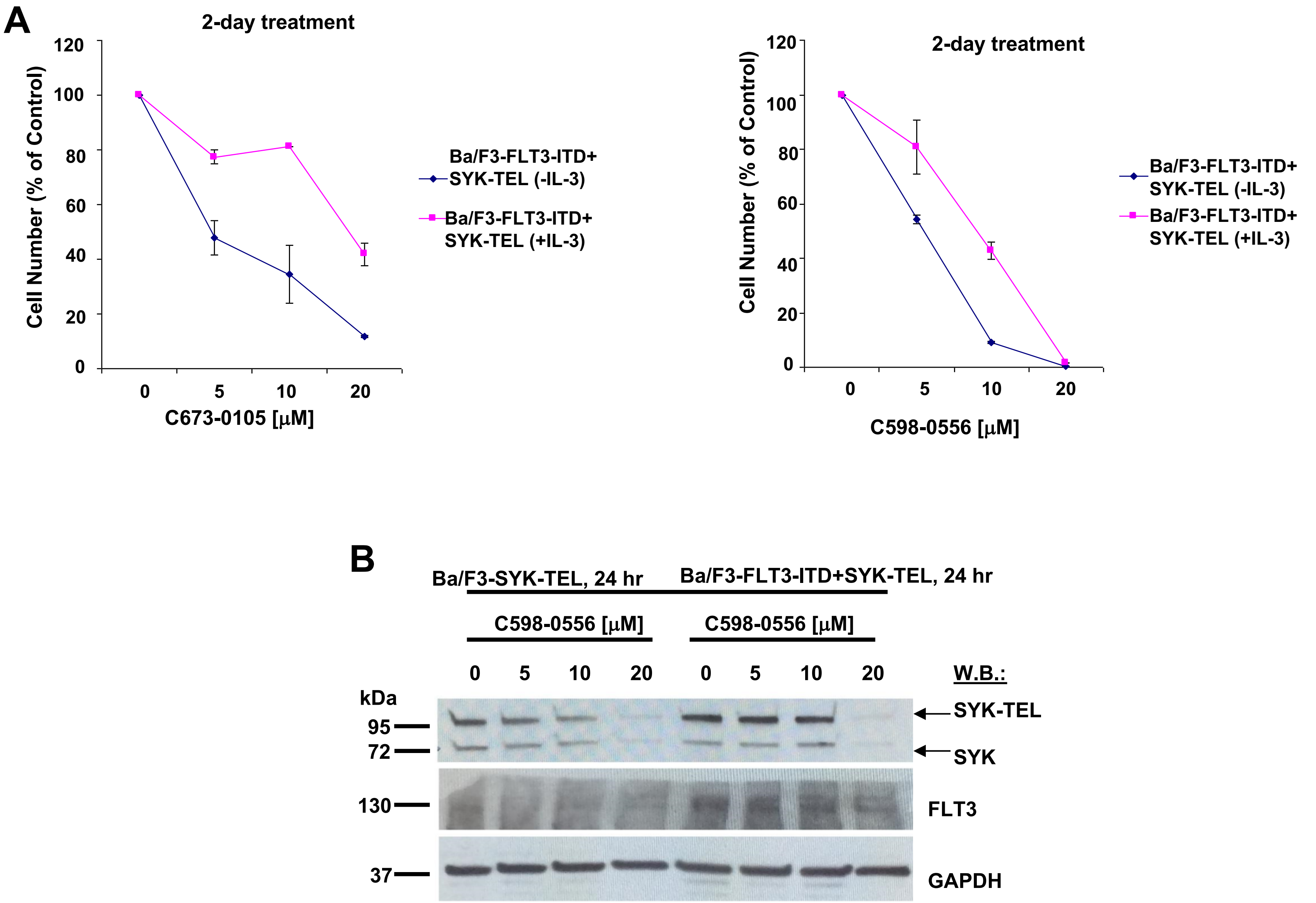

Supplementary Fig 9 (A-B)

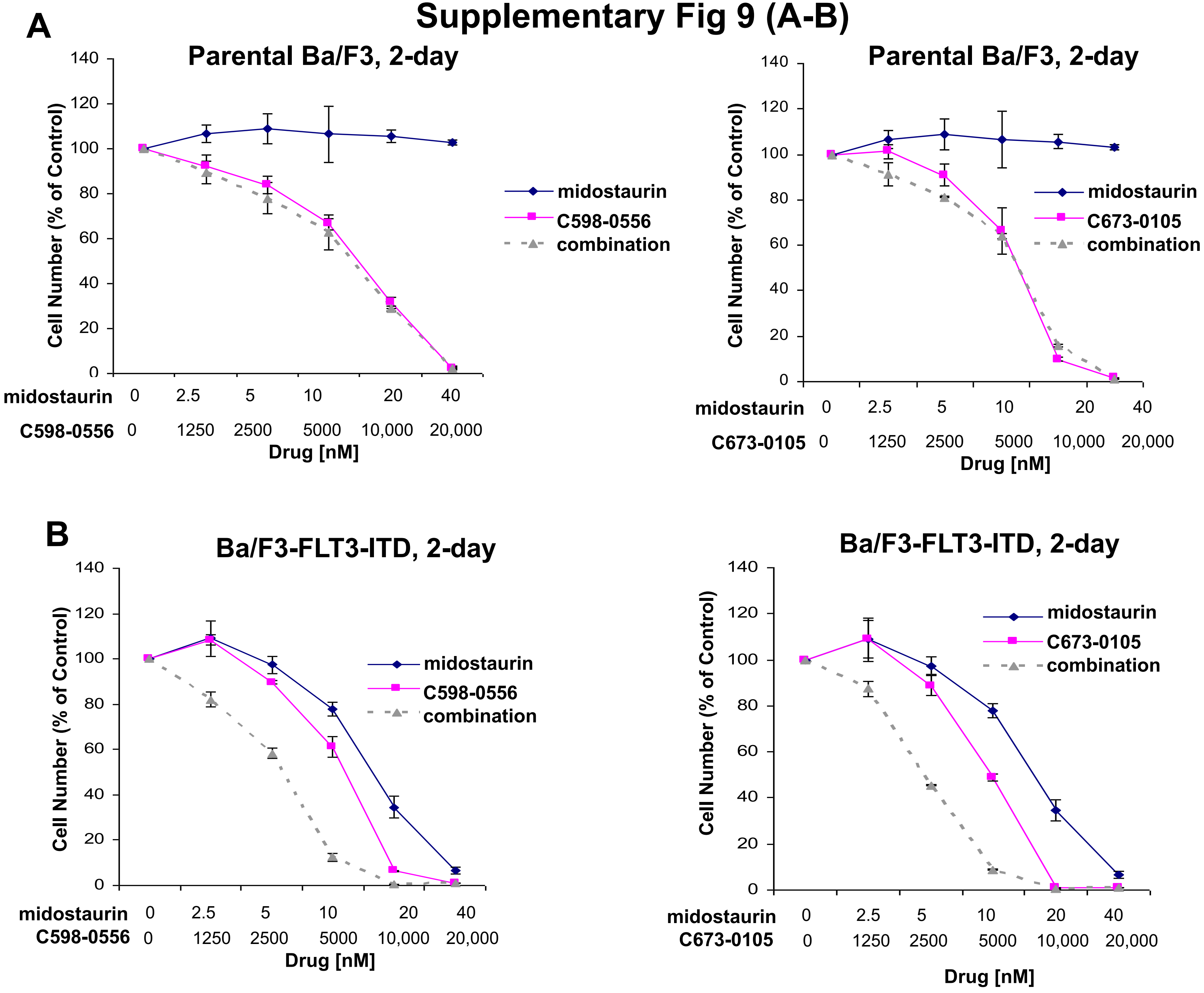

Supplementary Figure 9 (C)

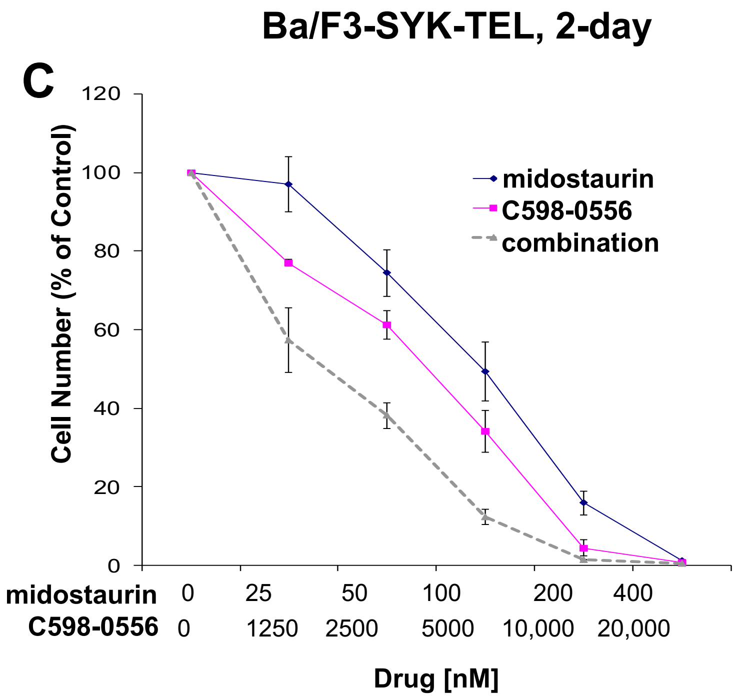

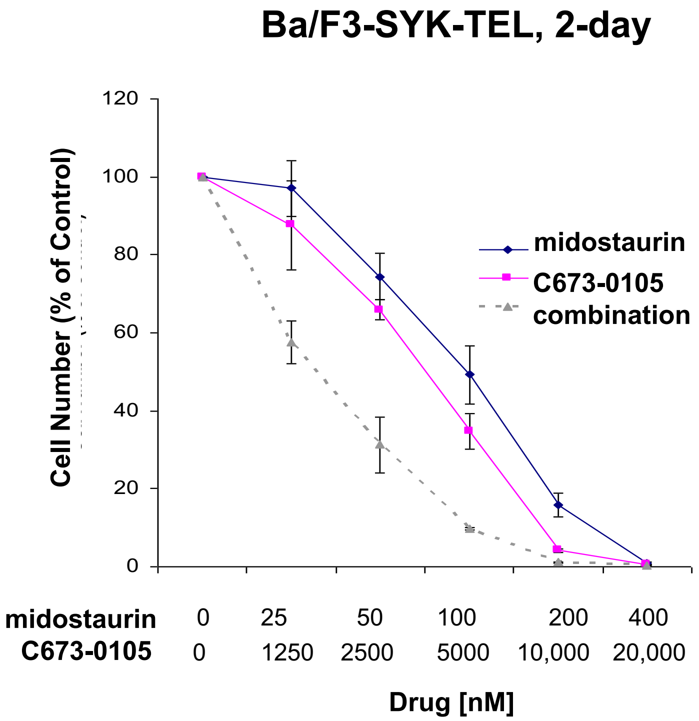

Supplementary Figure 9 (D-E)

D

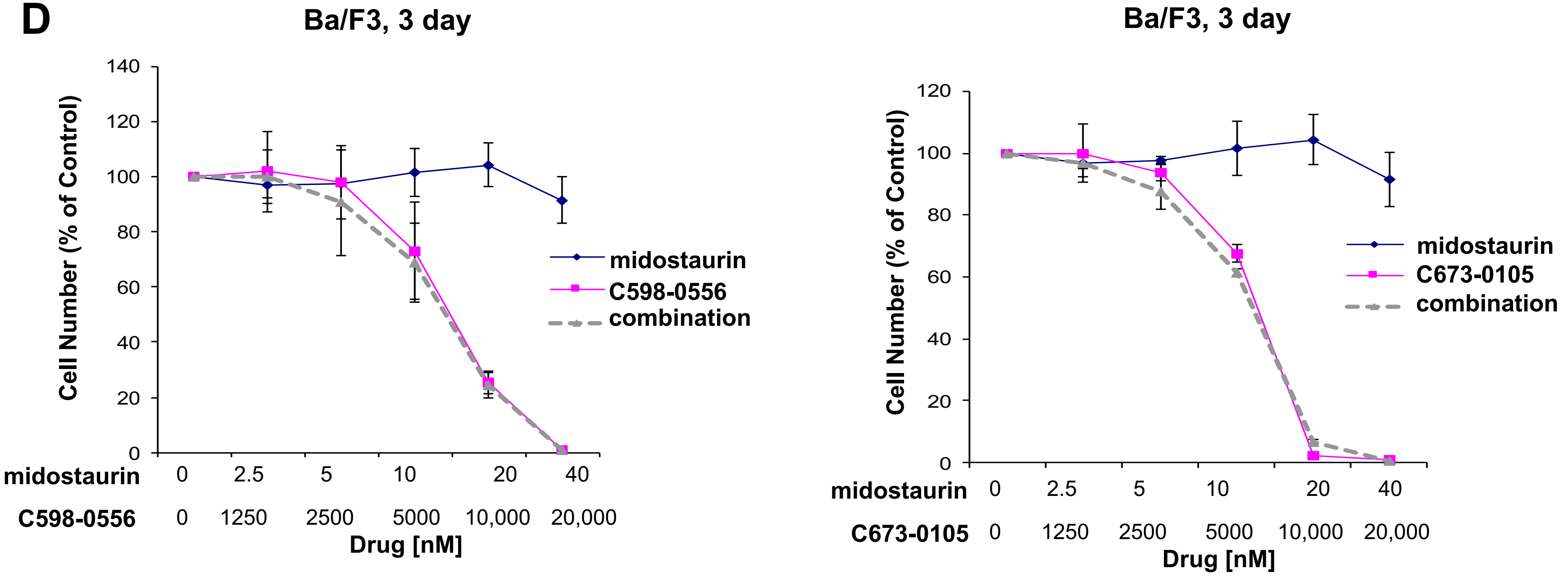

E

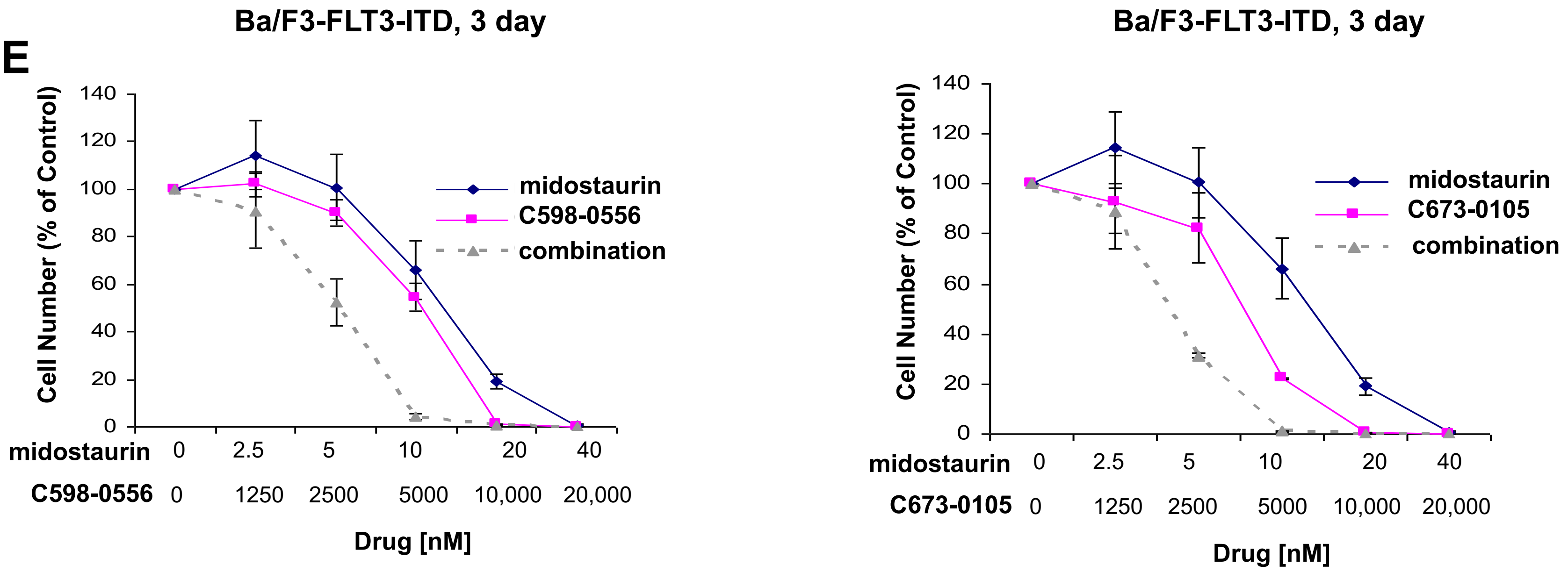

Supplementary Figure 9 (F-G)

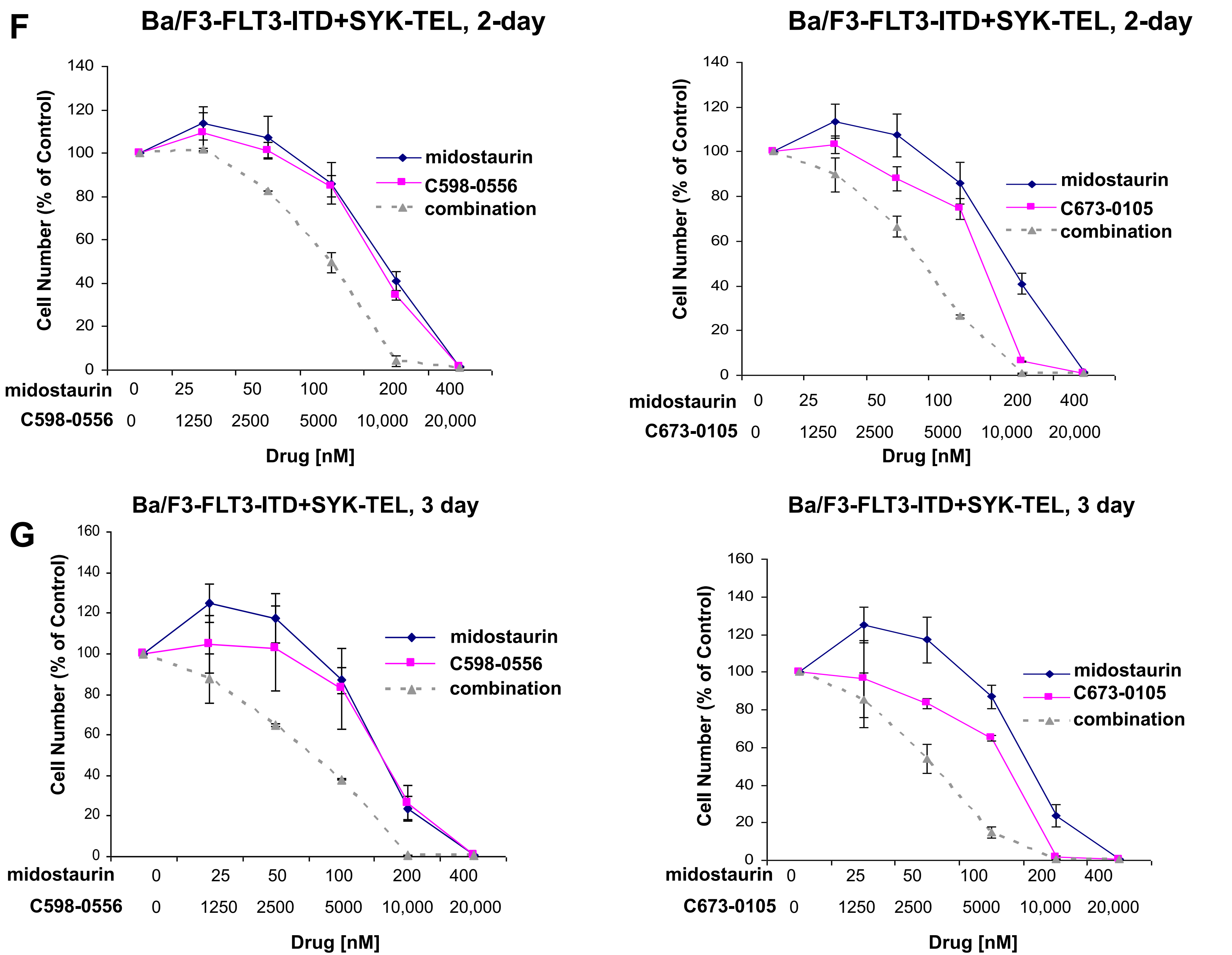

Supplementary Fig 9 (H-I)

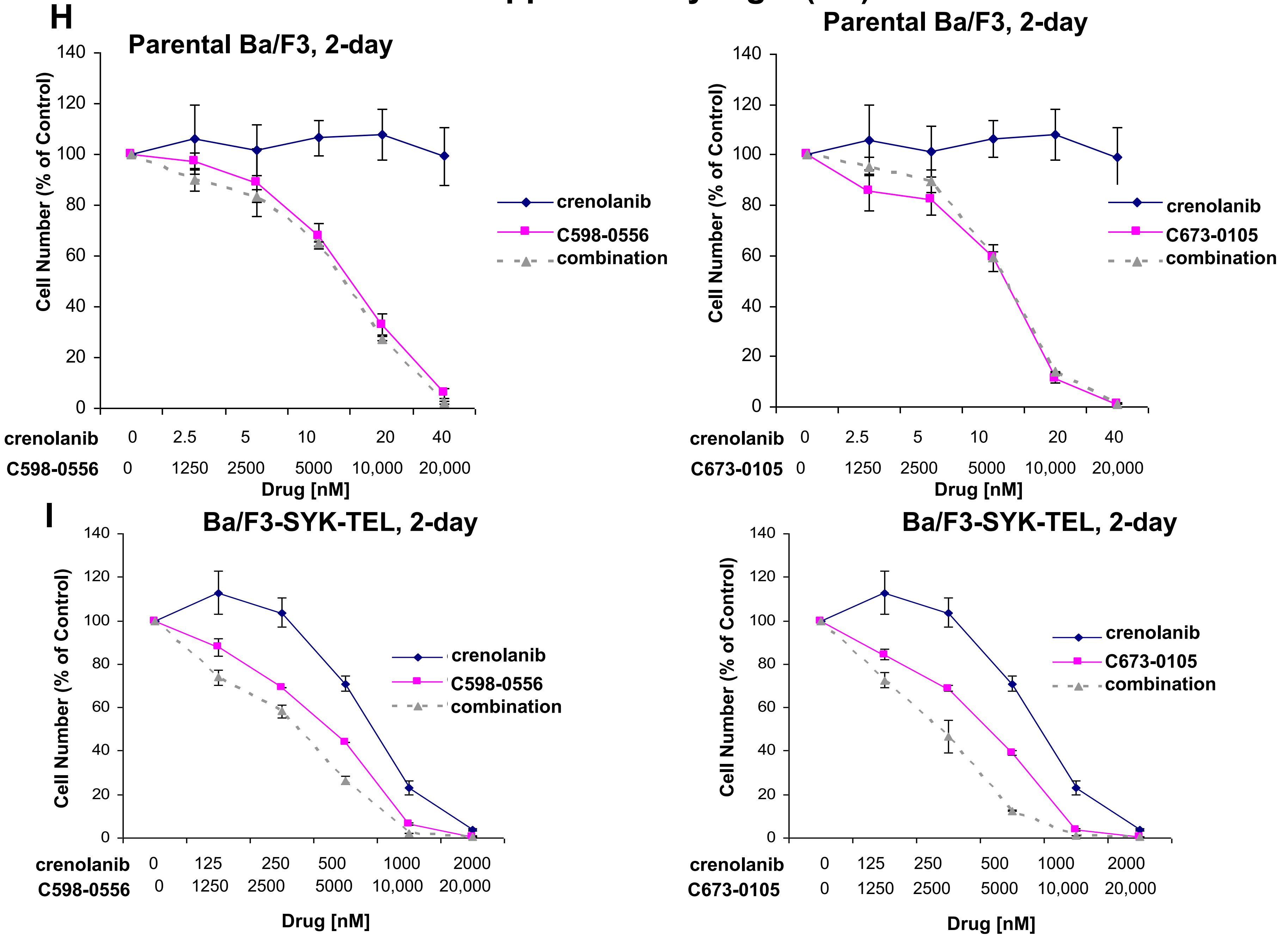

Supplementary Fig 9 (J-K)

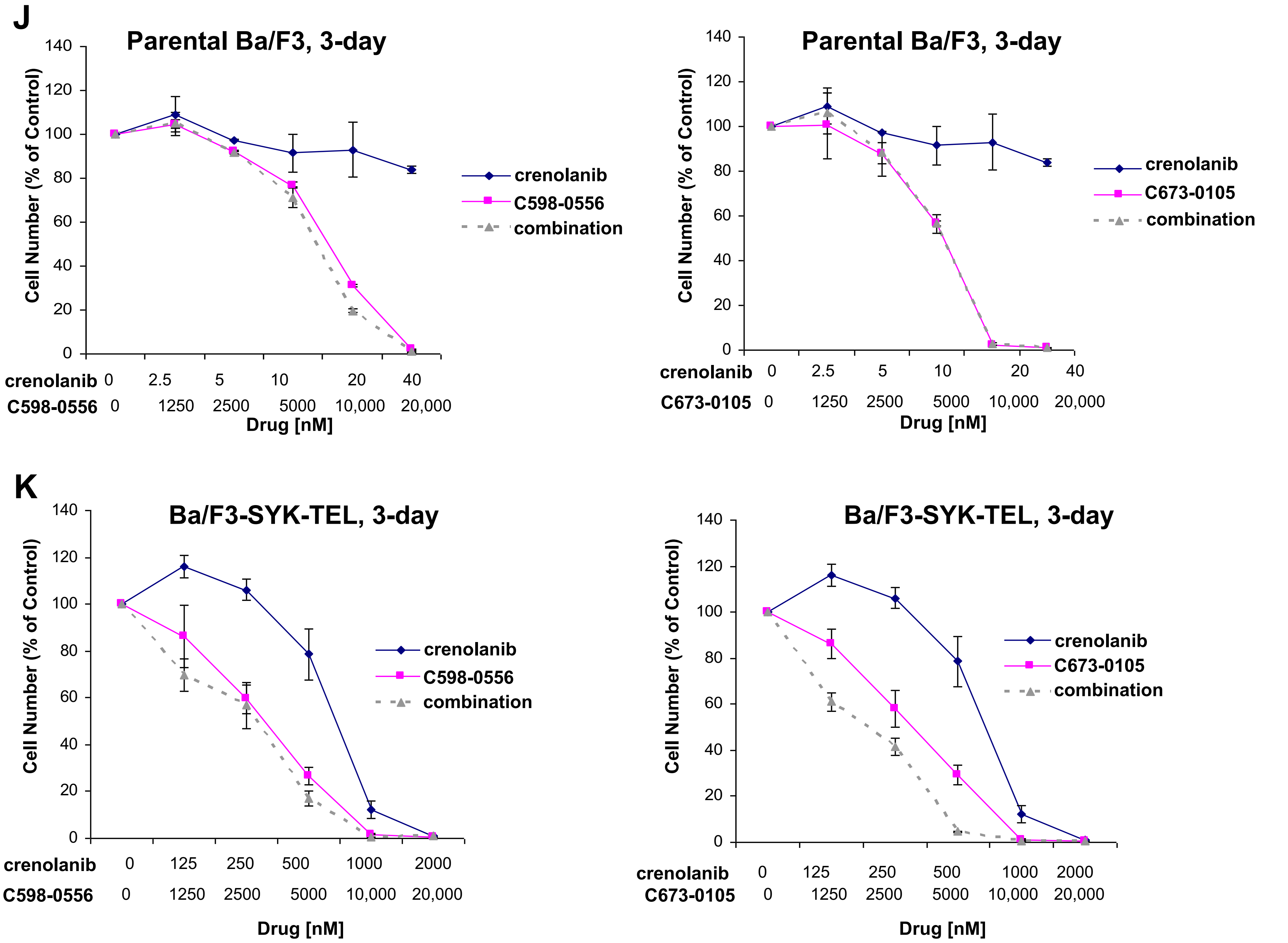

Supplementary Fig 9 (L-M)

L

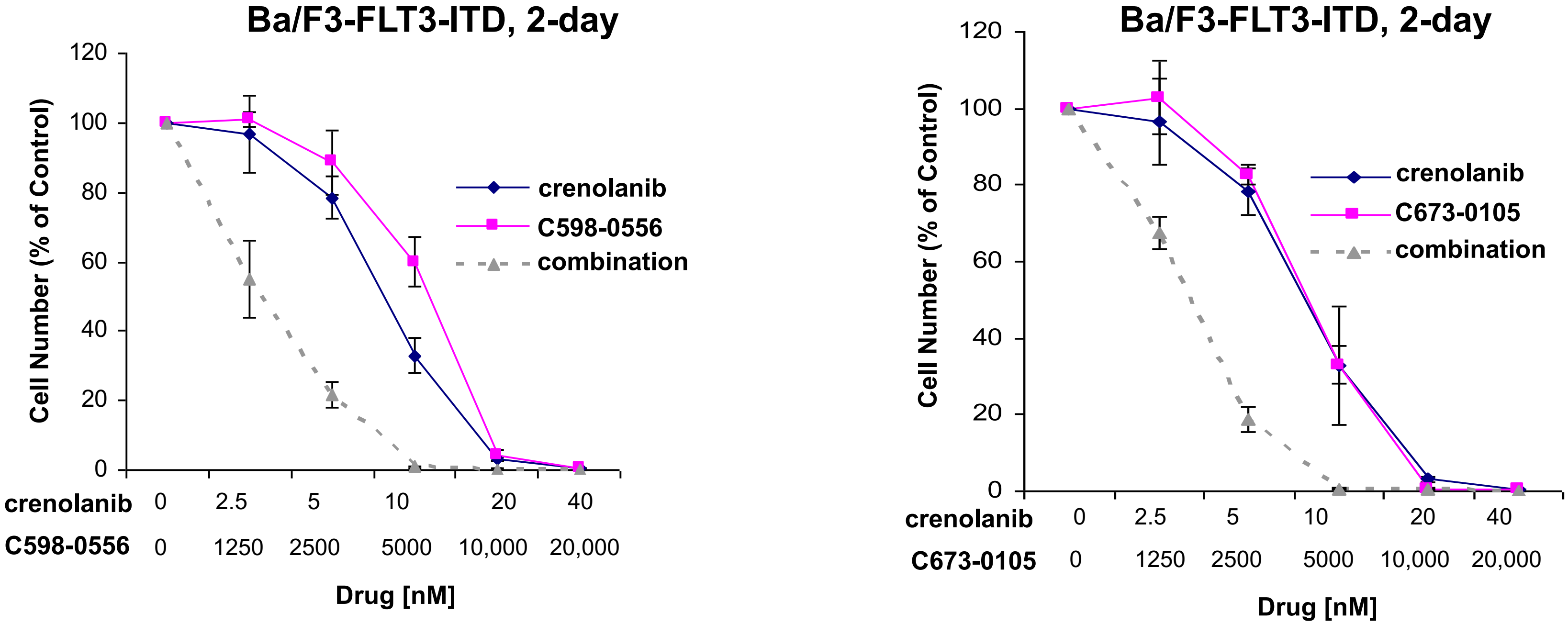

M

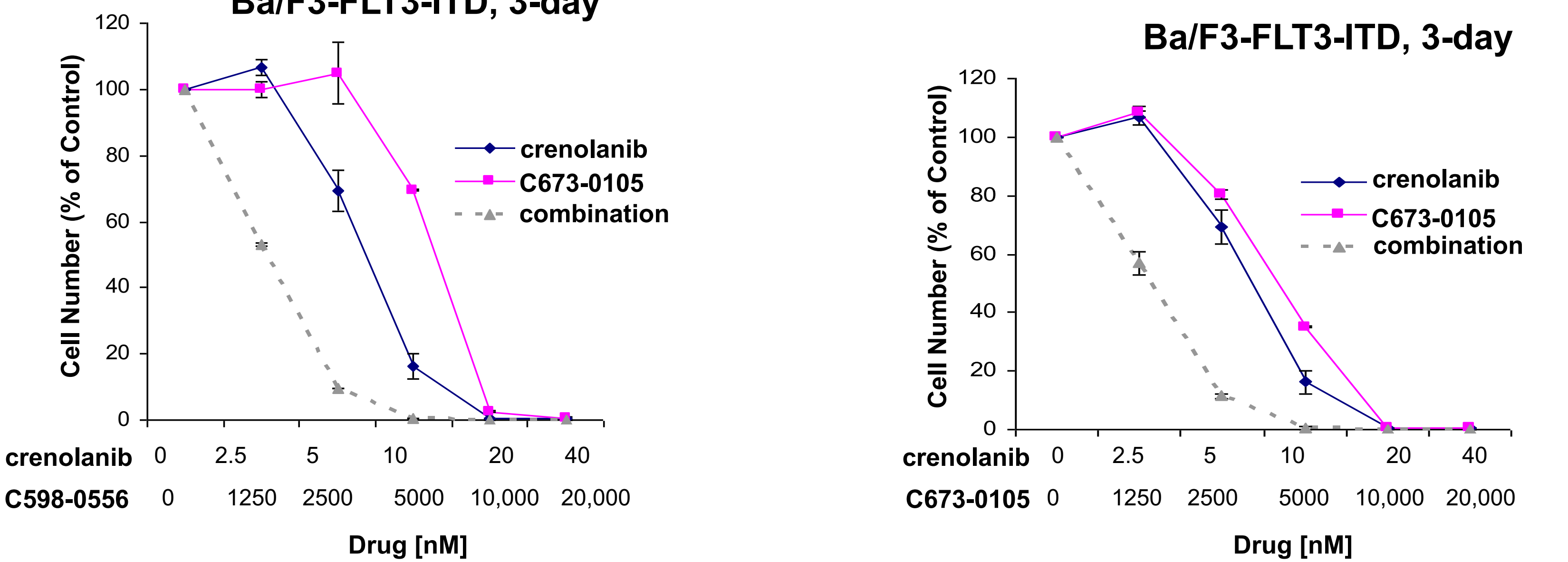

Supplementary Fig 9 (N-O)

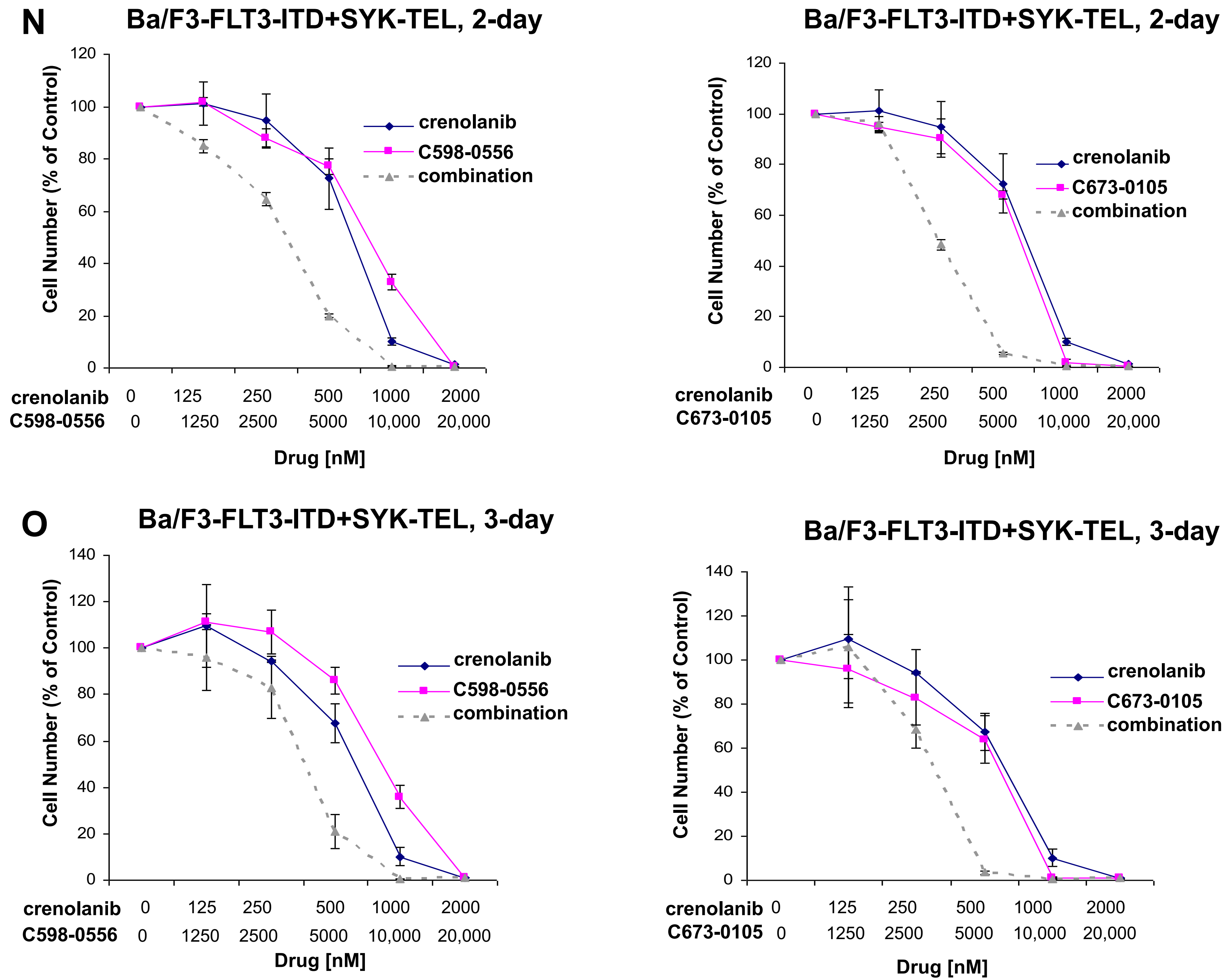

Supplementary Figure 1. P22077 induces degradation of SYK and FLT3 in FLT3-ITD-and TKD point mutant-expressing cells. (A, B) Immunoblots: 24 hr drug treatment of Ba/F3-FLT3-ITD+TKD point mutant cells.

Supplementary Figure 2. Induction of SYK degradation by USP10-targeting inhibitors. (A, B) Time course investigating the time of onset of SYK degradation in Ba/F3-SYK-TEL cells following treatment with C598-0556. (C-D) Induction of SYK degradation in Ba/F3-SYK-TEL cells by C598-0556. (E) Induction of FLT3 degradation in Ba/F3-FLT3-ITD cells by C598-0556, C598-0466, C598-0563, HBX19818, and P22077.

Supplementary Figure 3. Correlation between HBX19818 analog induced degradation of SYK and USP10 biochemical IC50s of HBX19818 analogs. (A) USP10 biochemical IC50s of HBX19818 and HBX19818 analogs using Ub-AMC as substrate; the ability of each compound to promote loss of FLT3 is indicated by + or – (Weisberg et al., 2017). (B) Immunoblots: Effects of 24 hours of treatment with C598-0563 on SYK and FLT3 protein levels in Ba/F3-FLT3-ITD-luc+ cells. (C) Immunoblot: Effects of 24 hours of treatment with C598-0563 on wt and activated SYK protein levels in Ba/F3-SYK-TEL cells.

Supplementary Figure 4. Physical association between USP10 and SYK and physical association between FLT3 and SYK. (A-B) Immunoprecipitation/immunoblots: Co-immunoprecipitation of SYK protein and USP10 protein in Ba/F3-SYK-TEL cells. (C) Purified GST or GST-SYK protein was incubated with His-USP10 coupled to NI-NTA beads. Proteins retained on beads were then blotted with indicated antibodies. (D)

Immunoprecipitation/immunoblot: Co-immunoprecipitation of FLT3 protein and SYK protein in Ba/F3-FLT3-ITD+SYK-TEL cells.

Supplementary Figure 5. Effects of USP10 or USP7 loss on SYK protein levels. (A) USP10 and USP7 Crispr KO in MOLM14-cas9-GFP #8 monoclonal cells. Shown in (A) are densitometry values for SCR versus T2 and T3, relative to GAPDH. (B) USP10 and USP7 Crispr KO in HEL-Cas9-GFP monoclonal cells.

Supplementary Figure 6. Genetic knockdown of USP10 leads to SYK degradation. (A-C) Immunoblots: Effects of USP10 KD on FLT3, SYK, MAPK and AKT expression in MOLM14 cells.

Supplementary Figure 7. USP10 KD shortens the half-life of SYK protein. (A,B) Effects of USP10 KD on the half-life of SYK in MOLM14 cells as compared to SCR control. This experiment is representative of two independent experiments for which similar results were observed (n=2). Shown are densitometry values for SCR versus shUSP10, shown relative to GAPDH.

Supplementary Figure 8. Small molecule inhibition of USP10 leads to targeted killing of Ba/F3-SYK-TEL and Ba/F3-FLT3-ITD+SYK-TEL cells and degradation of SYK. (A) Proliferation assay: 2-day treatment of Ba/F3-FLT3-ITD+SYK-TEL cells (+/-IL-3) with USP10-targeting inhibitors, C673-0105 (left panel) and C598-0556 (right panel). (B)

Immunoblots: Effects of 24 hours of treatment with C598-0556 on SYK protein levels in Ba/F3-SYK-TEL and Ba/F3-FLT3-ITD+SYK-TEL cells.

Supplementary Figure 9 (A-B). Midostaurin potentiates the effects of USP10 inhibitors against Ba/F3-FLT3-ITD cells, however not parental Ba/F3 cells (2 day assay). (A) Proliferation studies: Effects of the combination of midostaurin and C598-0556 (left panel) and midostaurin and C673-0105 (right) on growth of parental Ba/F3 cells following 2 days of treatment. (B) Proliferation studies: Effects of the combination of midostaurin and C598-0556 (left panel) and midostaurin and C673-0105 (right) on growth of Ba/F3-FLT3-ITD cells following 2 days of treatment.

Supplementary Figure 9 (C). Midostaurin potentiates the effects of USP10 inhibitors against Ba/F3-SYK-TEL cells (2 day assays). (C) Proliferation studies: Effects of the combination of midostaurin and C598-0556 (left panel) and midostaurin and C673-0105 (right) on growth of Ba/F3-SYK-TEL cells following 2 days of treatment.

Supplementary Figure 9 (D-E). Midostaurin potentiates the effects of USP10 inhibitors against Ba/F3-FLT3-ITD cells, however not parental Ba/F3 cells (3 day assays). (D) Proliferation studies: Effects of the combination of midostaurin and C598-0556 (left panel) and midostaurin and C673-0105 (right) on growth of Ba/F3 cells following 3 days of treatment. (E) Proliferation studies: Effects of combination of midostaurin and C598-

0556 (left panel) and midostaurin and C673-0105 (right panel) on growth of Ba/F3-FLT3-ITD cells following 3 days of treatment.

Supplementary Figure 9 (F-G). Midostaurin potentiates the effects of USP10 inhibitors against Ba/F3-FLT3-ITD+SYK-TEL cells (2, 3 day assays). (F) Proliferation studies: Effects of the combination of midostaurin and C598-0556 (left panel) and crenolanib and C673-0105 (right) on growth of Ba/F3-FLT3-ITD+SYK-TEL cells following 2 days of treatment. (G) Proliferation studies: Effects of combination of midostaurin and C598-0556 (left panel) and midostaurin and C673-0105 (right panel) on growth of Ba/F3-FLT3-ITD+SYK-TEL cells following 3 days of treatment.

Supplementary Figure 9 (H-I). Crenolanib potentiates the effects of USP10 inhibitors against Ba/F3-SYK-TEL cells, however not parental Ba/F3 cells (2 day assay). (H) Proliferation studies: Effects of the combination of crenolanib and C598-0556 (left panel) and crenolanib and C673-0105 (right) on growth of parental Ba/F3 cells following 2 days of treatment. (I) Proliferation studies: Effects of the combination of crenolanib and C598-0556 (left panel) and crenolanib and C673-0105 (right) on growth of Ba/F3-SYK-TEL cells following 2 days of treatment.

Supplementary Figure 9 (J-K). Crenolanib potentiates the effects of USP10 inhibitors against Ba/F3-SYK-TEL cells, however not parental Ba/F3 cells. (J) Proliferation study: Effects of the combination of crenolanib and C598-0556 (left panel) and crenolanib and

C673-0105 (right panel) on growth of Ba/F3 cells following 3 days of treatment. (K) Proliferation study: Effects of combination of crenolanib and C598-0556 (left panel) and crenolanib and C673-0105 (right panel) on growth of Ba/F3-SYK-TEL cells following 3 days of treatment.

Supplementary Figure 9 (L-M). Crenolanib potentiates the effects of USP10 inhibitors against Ba/F3-FLT3-ITD cells (2, 3 day assays). (L) Proliferation studies: Effects of the combination of crenolanib and C598-0556 (left panel) and crenolanib and C673-0105 (right) on growth of Ba/F3-FLT3-ITD cells following 2 days of treatment. (M) Proliferation studies: Effects of combination of crenolanib and C598-0556 (left panel) and crenolanib and C673-0105 (right panel) on growth of Ba/F3-FLT3-ITD cells following 3 days of treatment.

Supplementary Figure 9 (N-O). Crenolanib potentiates the effects of USP10 inhibitors against Ba/F3-FLT3-ITD+SYK-TEL cells (2, 3 day assays). (N) Proliferation studies: Effects of the combination of crenolanib and C598-0556 (left panel) and crenolanib and C673-0105 (right) on growth of Ba/F3-FLT3-ITD+SYK-TEL cells following 2 days of treatment. (O) Proliferation studies: Effects of combination of crenolanib and C598-0556 (left panel) and crenolanib and C673-0105 (right panel) on growth of Ba/F3-FLT3-ITD+SYK-TEL cells following 3 days of treatment.
